# Maize edible-legumes intercropping systems for enhancing agrobiodiversity and belowground ecosystem services

**DOI:** 10.1038/s41598-024-64138-w

**Published:** 2024-06-21

**Authors:** Abdul A. Jalloh, Daniel Munyao Mutyambai, Abdullahi Ahmed Yusuf, Sevgan Subramanian, Fathiya Khamis

**Affiliations:** 1 International Centre of Insect Physiology and Ecology, P.O. Box 30772-00100, Nairobi, Kenya; 2 Department of Zoology and Entomology, University of Pretoria, Private Bag x20 , Hatfield, Pretoria, South Africa; 3 Department of Life Sciences, South Eastern Kenya University, P.O Box 170-90200, Kitui, Kenya; 4 Forestry and Agricultural Biotechnology Institute, University of Pretoria, Private Bag x20, Hatfield, Pretoria, South Africa

**Keywords:** Crop diversification, Soil health, Microbial communities, Fungal and bacterial activity, Metabarcoding, Sustainable agriculture, Agroecology, Soil microbiology

## Abstract

Intensification of staple crops through conventional agricultural practices with chemical synthetic inputs has yielded positive outcomes in food security but with negative environmental impacts. Ecological intensification using cropping systems such as maize edible-legume intercropping (MLI) systems has the potential to enhance soil health, agrobiodiversity and significantly influence crop productivity. However, mechanisms underlying enhancement of biological soil health have not been well studied. This study investigated the shifts in rhizospheric soil and maize-root microbiomes and associated soil physico-chemical parameters in MLI systems of smallholder farms in comparison to maize-monoculture cropping systems (MMC). Maize-root and rhizospheric soil samples were collected from twenty-five farms each conditioned by MLI and MMC systems in eastern Kenya. Soil characteristics were assessed using Black oxidation and Walkley methods. High-throughput amplicon sequencing was employed to analyze fungal and bacterial communities, predicting their functional roles and diversity. The different MLI systems significantly impacted soil and maize-root microbial communities, resulting in distinct microbe sets. Specific fungal and bacterial genera and species were mainly influenced and enriched in the MLI systems (e.g., *Bionectria solani*, *Sarocladium zeae*, *Fusarium algeriense*, and *Acremonium persicinum* for fungi, and *Bradyrhizobium elkanii*, *Enterobacter roggenkampii*, *Pantoea dispersa* and *Mitsuaria chitosanitabida* for bacteria), which contribute to nutrient solubilization, decomposition, carbon utilization, plant protection, bio-insecticides/fertilizer production, and nitrogen fixation. Conversely, the MMC systems enriched phytopathogenic microbial species like *Sphingomonas leidyi* and *Alternaria argroxiphii*. Each MLI system exhibited a unique composition of fungal and bacterial communities that shape belowground biodiversity, notably affecting soil attributes, plant well-being, disease control, and agroecological services. Indeed, soil physico-chemical properties, including pH, nitrogen, organic carbon, phosphorus, and potassium were enriched in MLI compared to MMC cropping systems. Thus, diversification of agroecosystems with MLI systems enhances soil properties and shifts rhizosphere and maize-root microbiome in favor of ecologically important microbial communities.

## Introduction

In response to the demands and needs of an increasing global population, there has been a surge in agricultural intensification based on chemical synthetic inputs centered around staple food crops such as grains (like maize (*Zea mays*), rice (*Oryza sativa*), sorghum (*Sorghum bicolor*), and wheat (*Triticum aestivum*)) and leguminous (cowpea (*Vigna unguiculata*), pigeon pea (*Cajanus cajan*), common bean (*Phaseolus vulgaris*), green gram (*Vigna radiata*), and soybean (*Glycine max*)) crops^1^. Conventional agro-intensification has played a crucial role in boosting food security worldwide using modern techniques, but it has also increased greenhouse gas emissions, chemical fertilizer leakage, soil erosion, and biodiversity loss^2,3^. While agricultural intensification aims to enhance agricultural yields, it centered around conventional practices such as monocropping, increased usage of synthetic chemical inputs among others. Addressing these challenges require focusing on ecological intensification including crop diversification, emphasizing on environmental quality, and the role of belowground biodiversity^2–4^. One effective strategy of ecological intensification is intercropping, where two or more crops are cultivated simultaneously in the same field, offering numerous advantages^3–7^. Intercropping highlights utilization of resources relying on natural agroecological services, and synergies among plant species to improve the soil and crop yields, reduce environmental risks, and promote agricultural sustainability^2–4,7^. For example, grains like wheat or maize are often intercropped with legumes because they can fix nitrogen in the soil, benefiting the companion crops^4,8^.

Maize, a high-yielding C4 species, is a critical food and cash crop component of intercropping systems such as push–pull technology (PPT) and maize edible-legume intercropping (MLI) systems. Planting maize in strips alternating with C3 species like wheat or legumes such as soybean, pigeon peas, cowpea, and sesame (*Sesamum indicum*) allows for relay/strip intercropping^8,9^. Additionally, maize and other cereals can be intercropped in alternate rows and/or mixed randomly with other grains or legumes^8,10,11^. In sub-Saharan Africa (SSA), intercropping has been shown to play crucial role in climate change adaptation, weed and insect-pest management, and soil organic carbon concentration, leading to smart farming technologies^5,7,12^. Despite limitations such as reduced mechanization and reliance on labor, these cropping systems offer distinct advantages for smallholder farmers in developing countries where labor availability is not a constraint^10,13^. These cropping systems have gained popularity among smallholder farmers in SSA due to their positive impact on plant productivity and soil health^2,3,14–16^. Intercropping establishes a positive correlation between plant production, microbial abundance, and diversity, with microbes playing a significant role in nutrient availability and transfer to plants, enhancing overall plant health and soil fertility^13,17,18^.

Soil habitat is an ecosystem known for its diverse array of living organisms (especially microbes) and essential micro and/or macro nutrients^7,19–21^. The rhizosphere offers a favorable habitat for bacteria, fungi, and other organisms to thrive^4,17,18^. Further, the root microbiome significantly contributes to plant growth and health by facilitating nutrient uptake, improving stress tolerance, and protecting against pathogens. The diversity and abundance of soil and root microorganisms are influenced by the soil's biological, physical, and chemical properties^2,20,22,23^. The choice of plants for specific cropping systems also plays a significant role in determining the composition of microbes in the rhizosphere, plant root, and soil health by increasing or decreasing their abundance and diversity^20,24^. Agricultural practices such as crop rotation, intercropping, cover cropping, and PPT have been shown to increase the microbiome diversity and abundance and soil health by enhancing symbiotic and non-symbiotic beneficial microbial communities^4,17,25^. On the contrary, monoculture cropping systems contribute to the decline of soil biota and negatively impact soils by increasing erosion and plant pathogens^4,26^. The over-reliance on a single crop plant in monoculture systems can lead to insect-pest outbreaks, soil nutrient imbalances, and reduced resilience to ecological stressors. Understanding the impact of specific crop combinations on the dynamics of belowground microbiome, soil health, and its impact on crop yields is essential for designing and optimizing multispecies cropping systems, aligning with the broader goal of enhancing agricultural sustainability^4,7,13,27–29^.

This study, therefore, assessed the impact of MLI systems on rhizosphere and maize-root microbiome and soil physico-chemical properties within maize farming systems. We compared the maize-monoculture cropping (MMC) system with four different MLI systems (maize-black bean, maize-pigeon pea, maize-common bean, and maize-green gram). We hypothesized that conditioning soil with different MLI systems shifts soil physico-chemical properties and rhizosphere and maize-root microbiome in favor of ecologically important microbes compared to MMC. We further hypothesized that different MLI systems impacted the belowground microbes and soil physico-chemical properties differently. The metabarcoding approach employed in this study effectively identified the beneficial fungal and bacterial communities associated within MLI systems.

## Materials and methods

### Study site

**Figure 1:**
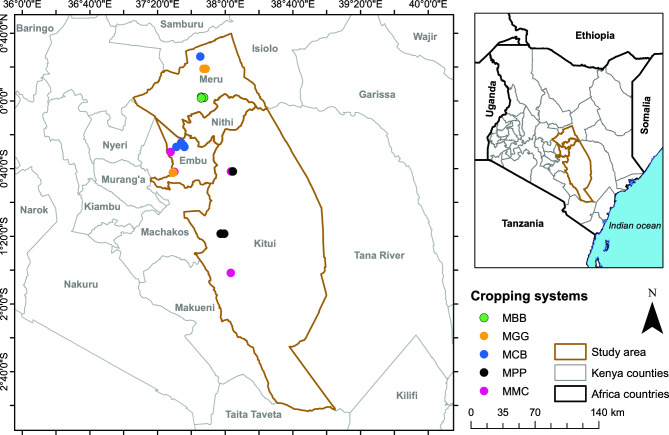
Map of Kenya showing maize edible-legume intercropping and maize-monoculture cropping systems in smallholder farms and counties where rhizospheric soil and maize*-*root samples were collected generated using QGIS, (2023) (v3.28.4); http://qgis.org.

The study was carried out in the following four counties in eastern Kenya: Tharaka Nithi (N 00° 01′ 58.5" E 37° 47′ 23.1"; N 00° 26′ 18.1" E 37° 45′ 19.8"; 700–1113 m above sea level (masl)), Embu (S 00° 30′ 07.4" E 37° 27′ 44.6; S 00° 31′ 08.1" E 37° 28′ 44.2"; 1093–1541 masl), Meru (N 00° 02′ 26.1" E 37° 45′ 55.5"; N 00° 01′ 48.6" E 37° 45′ 54.5"; 1110–1140 masl), and Kitui (S 00° 41′ 41.6" E 38° 3′ 30.7; S 01° 41′ 40.4" E 38° 3′ 21.1; 1085.32–1171.72 masl) (Fig. 1: QGIS, (2023) (v3.28.4); http://qgis.org). These counties have a hot and humid climate temperatures characterized by two distinct rainy seasons. Long rains occur from April to June, while shorter rains occur between November and December. Meru has a warm and cool climate with relatively dry weather. The average annual temperature in Meru ranges from 19.8 to 26.3 °C. Kitui has a tropical climate, with an average annual temperature of 21.9 °C. Embu's climate is warm and exhibits wet and dry periods, with an average yearly temperature of around 21.0 °C. Tharaka Nithi County has a savanna climate with distinct wet and dry seasons, with an average annual temperature of approximately 20.96 °C. These areas were chosen due to their established adoption of distinct cropping systems, encompassing maize edible-legume intercropping (MLI) and maize-monoculture (MMC) cropping systems^7,30^, which have been practiced for many years. These cropping systems were maintained with comparable agronomic practices with minimal weed management and pesticides and/or chemical fertilizers inputs. The MLI and MMC smallholder farms had ages ranging from 6 to 18 years and they depended entirely on seasonal rainfall.

### Sample collection

**Figure 2:**
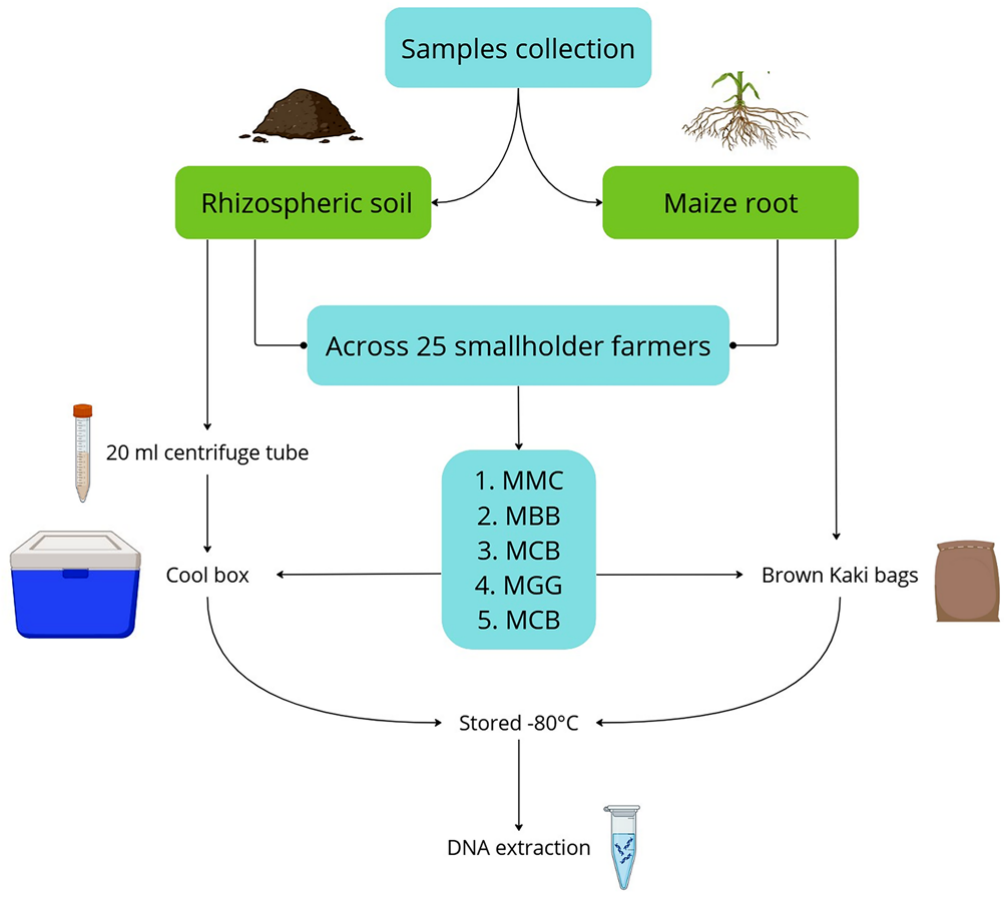
Method of sample collections from different cropping systems in smallholder farmer fields. MCB, Maize-common bean; MBB, maize-black bean; MGG, maize-green gram; MPP, maize-pigeon pea; and MMC, maize-monoculture cropping systems, figure generated using BioRender.com (2023) (https://app.biorender.com/).

Samples from MLI systems were collected from established smallholder farm fields representing different multiple cropping systems, namely maize-common bean (*Phaseolus vulgaris*) (MCB), maize-black bean (*Lablab purpureus*) (MBB), maize-green gram (*Vigna radiata*) (MGG), and maize-pigeon peas (*Cajanus cajan*) (MPP) intercrops (Fig. 2: BioRender.com (2023) (https://app.biorender.com/)). Rhizospheric soil and maize-root samples were collected from MLI and MMC cropping systems plots in the same farms or adjacent farms if the farm with MLI plots did not have an MMC plot. Sampling was conducted on twenty-five pairs of smallholder farms, five pairs per each MLI system when the maize plants were at vegetative stage (~ approximately six weeks old). Rhizospheric soil sampling was performed randomly between rows (maize edible-legume rows for MLI and maize-maize rows for MMC), with ten samples collected per farm at a 5–20 cm depth using a sterilized soil auger. Before sampling, the surface organic matter and debris around the rows were cleared. The rhizospheric soil samples were individually placed in 20 mL centrifuge tubes (Thermo Fisher Scientific Inc., California, USA) and stored in a cool box with ice packs. The collected samples were transported to the International Centre of Insect Physiology and Ecology (*icipe*) in Nairobi, Kenya, and stored at − 80 °C until DNA extraction. A subset of rhizospheric soil samples was also collected for physico-chemical analysis and kept in Brown Khaki Bags (Paper Bags Ltd., Nairobi, Kenya) for 48 h under room temperature before analysis.

For maize-root samples, ten plants adjacent to the soil cores were selected, and from each sampled plot, maize-root samples were collected from the root systems to a depth of approximately 10 cm. The collected maize-roots were rinsed to remove soil particles and subjected to surface sterilization using a 70% ethanol and 1% sodium hypochlorite solution for 1 min. The maize-roots were then washed six times with sterilized distilled water. Furthermore, to confirm sterility, 0.1 mL of the distilled water from the final rinsing stage was inoculated onto Lysogeny Broth, Potato Dextrose Agar, and Sabouraud Dextrose. The plates were then incubated at 33 ± 2 °C for 48 h and 25 ± 2 °C for 72 h to assess bacterial and fungal growth. After confirming sterility, the maize-root samples were air-dried with sterile blotting paper in an aseptic laminar flow hood, and later stored at − 80 °C awaiting DNA extraction.

### Genomic DNA extraction, PCR, library preparation, and sequencing

Total genomic DNA (gDNA) was extracted using the PureLink™ microbiome DNA purification kit (Thermo Fisher Scientific Inc., California, USA) using the manufacturer's protocols. This entailed the dual extraction of each (rhizospheric soil and maize-root) sample and the subsequent combination of the resulting supernatants to enhance the yield of DNA. After adding lysis buffer, power bead tubes holding 0.30 g of soil were vortexed for 10 s. The bead tubes were then vortexed for 5 min at 14,000 rpm after being heated at 65 °C for 10 min. Spin columns and Tris–HCL were used to extract the DNA. The DNA purity was evaluated using a Nanodrop 2000 UV–Vis spectrophotometer (Thermo Fisher Scientific Inc., California, USA) and through gel electrophoresis. The rhizospheric soil DNA samples were kept at − 80 °C^4,31^ until further downstream processing. Following 48 h freeze–drying process, maize-root samples were pulverized into a fine powder. DNA extraction from 0.30 g of the dried powdered maize-root was performed using the isolate II plant DNA extraction kit (Thermo Fisher Scientific Inc., California, USA). The finely powdered maize-root was placed within bead tubes containing lysis buffer and subjected to 30 s vortexing followed by 2 min of centrifugation at 14,000 rpm to extract the DNA using spin columns. The purity of the resultant maize-root DNA was evaluated (Thermo Scientific Nanodrop 2000 (UV–Vis spectrophotometer)) and stored as described above^4^.

Following the manufacturer's protocol, the metabarcoding amplicon was sequenced at Macrogen Europe (Netherlands) using the MiSeq Illumina system. 16S rDNA libraries were generated from the bacterial V3–V4 gene regions and ITS libraries from the fungal ITS1–ITS2 gene regions, using markers outlined in Table 1^4,32^. The library kit employed was the Herculase II Fusion DNA Polymerase Nextera XT Index V2 Kit. The libraries were sequenced in paired-end mode.

**Table 1:** PCR-specific primers/markers used for bacterial and fungal metagenomic amplicon sequencing.

PCR primers for 16S rDNA (bacterial) gene regions	
27F′	GAGTTTGATCMTGGCTCAG
338R′	AGTGCTGCCTCCCGTAGGAGT
341F′	CCTACGGGNGGWGCAG
805R′	GACTACHVGGGTATCTAATCC
PCR primers for ITS1-ITS2 gene regions (fungal)	
ITS1F′	CTTGGTCATTTAGAGGAAGTAA
ITS2R′	GCTGCGTTCTTCATCGATGC
ITS3F′	GCATCGATGAAGAACGCAGC
ITS4R′	TCCTCCGCTTATTGATAGC

### Bioinformatics analyses

The nf-core/ampliseq (v2.4.0) metagenomic amplicon pipeline, implemented using nextflow (v21.10.3) and singularity (v3.6.3), was used to process the raw reads from rhizospheric soil and maize-root samples^2,4^. The Divisive Amplicon Denoising Algorithm 2 (DADA2, v1.26.0) analysis workflow option was utilized to generated maize-root and rhizospheric soil mycobiome and prokaryotic population community data, including the amplicon sequence variant (ASVs) abundance tables and taxonomic classification. Data exploration, statistical analysis, and visualization were performed using R packages (v4.2.1). Quality control was assessed using FastQC, and poor reads and primers were trimmed using Cutadapt (v4.1). The DADA2 was employed for denoising, pre-processing, inferring ASVs, and assigning taxonomy based on clean sequence reads. Dereplication, ASV inference, and chimera removal steps were executed using specific DADA2 functions^2,27^. Taxonomy assignment utilized the “Silva = 138” and “unite-Fungi = 8.3” databases for 16S rDNA (bacterial) and ITS (fungal) ASVs, respectively^33^. The ASV nucleotide base sequences were classified using Basic Rapid Ribosomal RNA Predictor (Barrnap, v0.9) into several groups of mycobiome, eukaryotes, bacteria, and prokaryotic, among others^34^. However, archaea, eukaryotes, chloroplasts, and mitochondria were excluded during ASV filtering of bacterial, while for fungal, archaea, chloroplasts, and mitochondria were removed during ASV filtering^4,35^. The resulting microbial communities of the ASV abundance matrix, taxonomy table, and metadata were merged into a phyloseq object^36^. Differential abundance analysis was conducted using the phyloseq (v1.41) package to assess alpha and beta diversity^37^. The metagMisc (v.0.04) package^38^, facilitated manipulation and visualization of the phyloseq object, presenting microbiome relative abundances and percentages across sample types (maize-root and rhizospheric soil), cropping systems (MMC and MLI (MBB, MPP, MCB, and MGG)), and sample locations (Embu, Meru, Tharaka Nithi, and Kitui counties).

To assess genus and species richness and diversity of the mycobiome and prokaryotic sequences, the Microbiota Process function (v1.9.3) performed rarefaction analysis based on ASV read counts. Alpha diversity metrics were utilized, including *Chao1*, *Evenness*, and *Shannon* indices^39^. A Principal Coordinate Analysis (PCoA) was conducted to identify fungal and bacterial contributors to beta diversity variation. The shared microbial communities among sample types, cropping systems, and sampling locations were visualized using Venn diagrams generated with the Venn Counts function in the limma package (linear models for microarray data)^2,40^. Permutational Multivariate Analysis of Variance (PERMANOVA), with each smallholder farm being treated as strata, was employed to compare fungal and bacterial populations across cropping systems, sample types, and studied locations. The phyloseq object file was used to determine biomarkers and visualize fungi and bacteria at the genera and species levels using diff_analysis and ggdiffclade functions of the Microbiota Process^41^.

### Soil physico-chemical analyses

Physico-chemical parameters of soil samples collected from the various MLI and MMC cropping systems were analyzed for micronutrients, soil texture, and trace elements at Société Générale de Surveillance (SGS) Kenya Ltd., Multi-laboratory, Nairobi, according to the established protocols described by Okalebo et al.^42^.

### Statistical analyses

The Shapiro–Wilk test was used to evaluate the uniformity of soil physico-chemical properties data, which was normally distributed. One-way analysis of variance (ANOVA) with the Newman Keuls test was used to analyze the soil physico-chemical properties conditioned by the various MLI and MMC cropping systems^7^. Tukey HSD test was used to determine significant differences of soil physico-chemical properties. In order to investigate the connections between the soil conditions under the different cropping systems and the shifts in soil physico-chemical properties among MLI and MMC cropping systems soil, non-multidimensional scaling (NMDS) using Bray–Curtis dissimilarity matrix was used^39^. All statistical analyses were performed using R software v4.2.1^38^.

## Results

### Composition, relative abundance, and taxonomic profiles of fungal and bacterial microbiome in rhizospheric soil and maize-root from different cropping systems

Through ITS community profiling, a total of 1,903,882 high-quality reads was acquired, with an average of 47,597 reads per sample and a range of 71,152 and 27,586. While, for 16S community 1,657,160 reads were obtained, with each sample containing between 51,915 and 28,256 reads, with a mean of 37,662 reads. After excluding non-bacterial and non-fungal sequence reads and conducting the rarefaction process, 712,831 fungal and 704,627 bacterial amplicon sequence variants (ASVs) were detected through all samples from both maize edible-legume intercropping (MLI (MBB, MGG, MCB, and MPP)) and maize-monoculture (MMC) cropping systems (including rhizospheric soil and maize-root samples).

Striking differences between crop diversification on MLI and MMC cropping systems among fungal and bacterial communities were evident in the ASV’s relative abundance across various study locations, sample types, and cropping systems. The MLI systems exhibited distinct microbial habitats with specific sets of fungi and bacteria. Within the fungal genera communities, *Setophoma* (23.40%) was most relatively abundant in MBB soil, *Epicoccum* (26.60%) dominated MCB soil, and *Cladosporium* (22.00%, and 9.50%) were more prevalent in MGG and MPP soil, respectively (Fig. S1A; Table S1). In MMC soil, *Alternaria* (38.50%) was the dominant genus. In maize-root samples, *Setophoma* (53.60%) prevailed in MBB, *Cladosporium* (41.70%) in MCB, *Fusarium* (37.50%) in MPP, and *Setophoma* (56.00%) in MMC maize-root (Fig. S1B; Table S2). Moreover, a similar trend on relative abundance rankings of fungal genera in study locations, cropping systems, and sample types are detailed in the supplementary results, Fig. S1C; Table S3.

**Figure 3:**
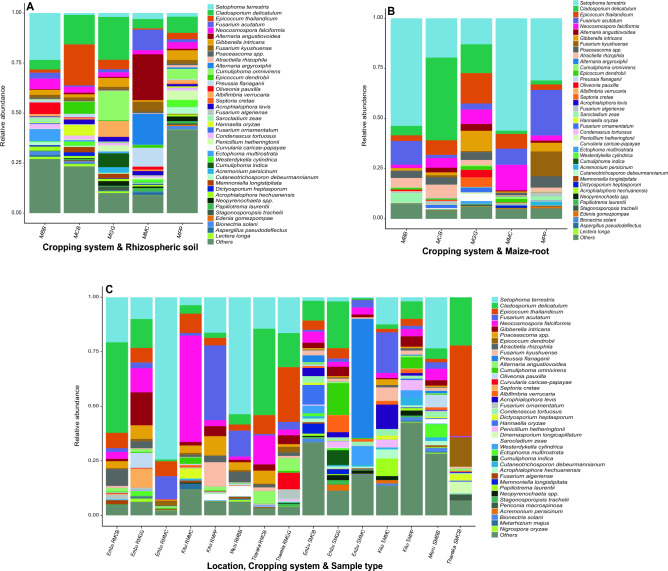
Relative abundance of fungal species. (**A**) cropping systems and rhizospheric soil samples; (**B**) cropping systems and maize-root samples; (**C**) study locations, cropping systems and sample types. R, maize-root; S, rhizospheric soil; MPP, maize-pigeon pea; MBB, maize-black bean; MCB, maize-common bean; MGG, maize-green gram; and MMC, maize-monoculture cropping systems. Fungal species with relative abundance lower than 1% were grouped as others.

MLI soils exhibited a higher diversity of fungal species than MMC soils. *Setophoma terrestris* (23.40%) was most relatively abundant in MBB soil, while *Epicoccum thailandium* (20.50%) dominated MCB. *Cladosporium delicatulum* (21.40%, and 8.10%) were relatively abundant in both MGG and MPP soils, whereas *Alternaria angustiovoidea* (23.00%) was more enriched in MMC soil (Fig. 3A; Table S4). In maize-root samples, *Setophoma terrestris* were predominant in MMC (56.00%), MMB (53.60%), and MPP (30.90%) maize-roots, while *Cladosporium delicatulum* (41.30%) dominated MCB, and *Epicoccum thailandicum* (15.20%) were prevalent in MGG maize-roots (Fig. 3B; Table S5). Analyzing interactions between fungal species across study locations, cropping systems, and sample types (R for maize-root samples and S for rhizospheric soil samples), *Cladosporium delicatulum* (41.60%) was enriched in Embu RMCB. *Gibberella intricans* (15.10%) dominated Embu RMGG. *Setophoma terrestris* (74.40%, and 53.60%) was more prevalent in Embu RMMC and Meru RMBB. *Neocosmospora falciformis* (48.70%) was abundant in Kitui RMMC, and *Fusarium acutatum* (34.30%, and 18.70%) dominated Kitui RMPP and Kitui SMMC, respectively. In Tharaka Nithi RMGG and SMCB, *Epicoccum thailandicum* (25.10%, and 41.40%) were prevalent. In Embu SMMC, *Preussia flanaganii* (54.60%) dominated (Fig. 3C; Table S6).

The most relatively abundant bacterial genera in MLI systems compared to MMC cropping systems soils were as follows: *Bacillus* were more abundant (MPP, 51.50%; MMC, 46.90%; MBB, 45.60%; and MPP, 26.40%). *Bradyrhizobium* showed more relative abundance in MGG (88.90%) (Fig. S2A; Table S7). In maize-root samples, *Ralstonia* (37.20%) was relatively abundant in MBB, *Bacillus* (21.30%) in MCB, and *Bradyrhizobium* (74.70%, and 41.00%) in MGG and MMC, while *Mitsuaria* (20.40%) was prevalent in MPP maize-root (Fig. S2B; Table S8). Similar trend in the relative abundance rankings of bacterial genera in study locations, cropping systems, and sample types were observed as provided in the supplementary results, Fig. S2C; Table S9.

**Figure 4:**
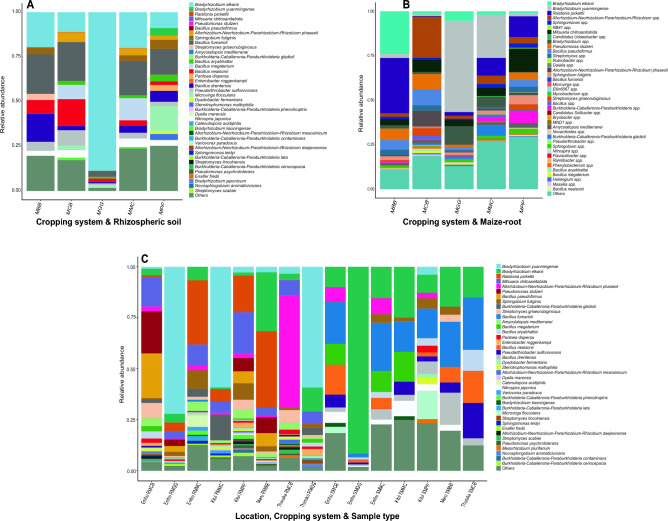
Relative abundance of bacterial species. (**A**) cropping systems and rhizospheric soil samples; (**B**) cropping systems and maize-root samples; (**C**) study locations, cropping systems and sample types. R, maize-root; S, rhizospheric soil; MPP, maize-pigeon pea; MBB, maize-black bean; MCB, maize-common bean; MGG, maize-green gram; and MMC, maize-monoculture cropping systems. Bacterial species with relative abundance lower than 1% were grouped as others.

A similar pattern was observed in bacterial species in cropping systems and soil types, where the most relatively abundant species in MBB, MCB, and MPP were *Bacillus fumarioli* (22.10%, 21.70%, and 14.50%). *Bradyrhizobium elkanii* (88.90%, and 20.10%) dominated MGG and MMC soil, respectively (Fig. 4A; Table S10). In maize-root samples, *Ralstonia pickettii* (37.20%) prevailed in MBB, *Bacillus pseudofirmus* (16.3%) in MCB, and *Bradyrhizobium yuanmingense* in MGG (67.40%) and MMC (37.6%) respectively, while *Mitsuaria chitosanitabida* (20.40%) was relatively abundant in MPP (Fig. 4B; Table S11).

The interactions between bacterial species across study locations, cropping systems, and sample types (R for maize-root samples and S for rhizospheric soil samples), *Bacillus pseudofirmus* (22.00%) was enriched in Embu RMCM, *Bradyrhizobium yuanmingense* (15.10%) in Embu RMGG, and *Ralstonia picketti* (31.40%) in Embu RMMC and Meru RMBB, respectively. *Bradyrhizobium yuanmingense* (59.10%, and 69.20%) dominated Kitui RMMC and Tharaka Nithi RMGG. *Bacillus fumarioli* (25.70%, 23.60%, 22.10%, 20.60%, and 14.50%) were the most highly relative abundance species in Embu SMGG, Meru SMBB, Kitui SMMC, Kitui SMPP, and Tharaka Nithi SMCB. However, in Kitui RMPP, *Mitsuaria chitosanitabida* (20.40%) dominated (Fig. 4C; Table S12).

### Alpha diversity of rhizospheric soil and maize-root fungal community in different maize edible-legume intercropping and maize-monoculture cropping systems

**Figure 5:**
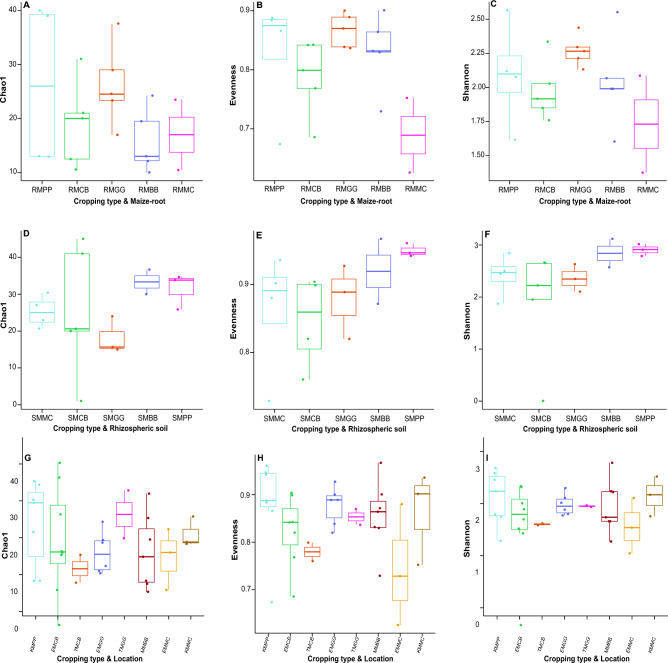
Alpha diversity of fungal species communities. (**A, B, C**) cropping systems and maize-root (R) samples; MPP, maize-pigeon pea; MBB, maize-black bean; MCB, maize-common bean; MGG, maize-green gram; and MMC, maize-monoculture cropping systems; (**D, E, F**) cropping systems and rhizospheric soil (S) samples; (**G, H, I**) study location and copping systems; KMPP, Kitui MPP; EMCB, Embu MCB; TMCB, Tharaka Nithi MCB; EMGG, Embu MGG; TMGG, Tharaka Nithi MGG; MMBB, Meru MBB; EMMC, Embu EMMC; KMMC, Kitui MMC.

There was no significant difference in fungal species communities between different MLI and MMC cropping systems in terms of richness and evenness (Fig. S3; *Chao1* and *Shannon* index estimator *P* values: see the full list, Tables S13 and S14). No significant difference in fungal species communities was observed in both cropping systems (MLI and MMC) and sample types (rhizospheric soil (S) + maize-root (R)) through richness and evenness. (Fig. 5; *Chao1* and *Shannon* index estimator *P* values: see the full list, Table S15). However, a significant difference was observed in richness and evenness when considering the interaction between cropping systems and sample types (*Chao1* estimator: RMBB vs RMGG *P* = 0.056; SMCB vs SMMC *P* = 0.02, and *Shannon* index estimator: SMCB vs SMPP *P* = 0.036). There was no significant difference in fungal species communities between cropping systems (MLI and MMC) and study locations (Embu (E), Kitui (K), Tharaka Nithi (T), and Meru (M)) in terms of richness and evenness, (Fig. 5; *Chao1* and *Shannon *index estimator *P* values: see the full list, Table S16).

### Alpha diversity of rhizospheric soil and maize-root bacterial community in maize edible-legume intercropping and maize-monoculture cropping systems

**Figure 6:**
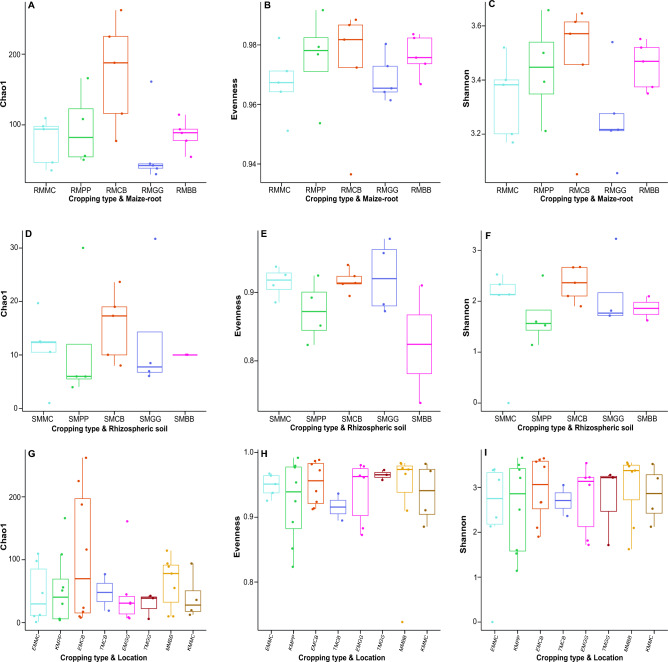
Alpha diversity of bacterial species communities. (**A, B, C**) cropping systems and maize-root (R) samples; MPP, maize-pigeon pea; MBB, maize-black bean; MCB, maize-common bean; MGG, maize-green gram; and MMC, maize-monoculture; cropping systems; (**D, E, F**) cropping systems and rhizospheric soil (S) samples; and (**G, H, I**) study location and copping systems; KMPP, Kitui MPP; EMCB, Embu MCB; TMCB, Tharaka Nithi MCB; EMGG, Embu MGG; TMGG, Tharaka Nithi MGG; MMBB, Meru MBB; EMMC, Embu EMMC; KMMC, Kitui MMC.

There was no significant difference in bacterial species communities between MLI and MMC cropping systems through assessment of the richness and evenness (Fig. S4; *Chao1* and *Shannon* index estimator *P* values: see the full list, Tables S17 and S18). Similarly, there were no significant differences in bacterial species communities between cropping systems (MLI and MMC) and sample types (rhizospheric soil (S) + maize-root (R)) in terms of richness and evenness (Fig. 6; *Chao1* and *Shannon* index estimator *P* values: see full list; Table S19). However, a significant difference was observed in richness and evenness when considering the interaction between some cropping systems and samples types (Fig. 6; *Chao1* estimator: RMCB vs RMGG *P* = 0.032; RMCB vs RMMC *P* = 0.056, RMGG vs RMMC *P* = 0.056 and, and *Shannon* index estimator: SMMC vs SMPP *P* = 0.056). The *Chao1* and *Shannon* index estimator also demonstrated no significant differences among bacterial species communities between cropping systems (MLI and MMC) and study locations (Embu (E), Kitui (K), Tharaka Nithi (T) and Meru (M)) in terms of richness and evenness, (Fig. 6; *P* values: see the full list, Table S20).

### *Beta* diversity and the effect of maize edible-legume intercropping and maize-monoculture cropping systems on rhizospheric soil and maize-root microbiomes

**Figure 7:**
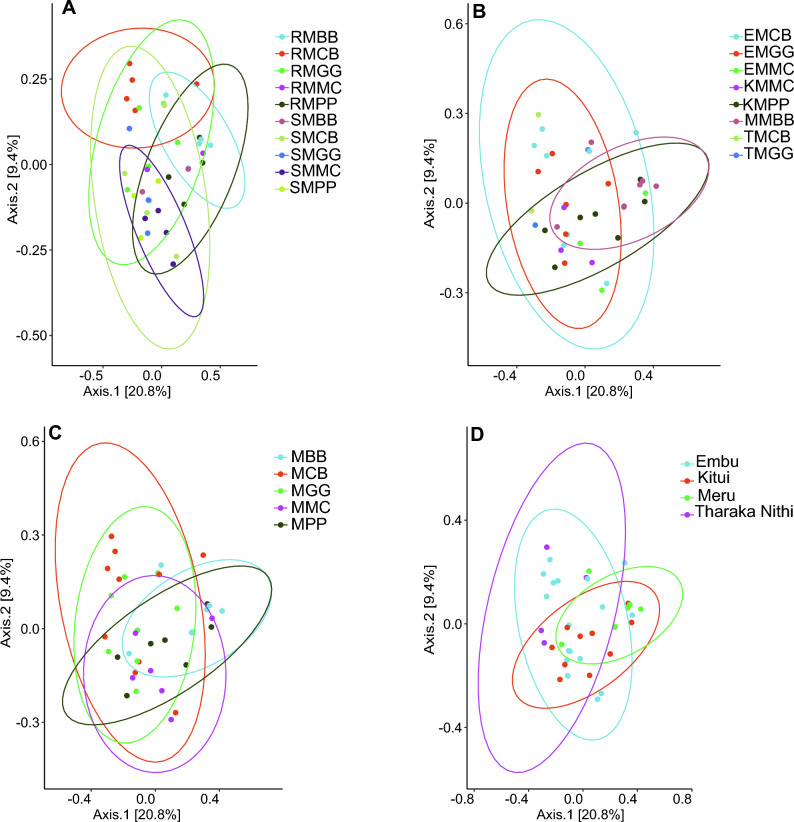
Beta diversity of fungal species communities. (**A**) cropping systems and sample types; maize-root (R), and rhizospheric soil (S) samples; MPP, maize-pigeon pea; MBB, maize-black bean; MCB, maize-common bean; MGG, maize-green gram; and MMC, maize-monoculture cropping systems; (**B**) cropping systems and study locations; EMCB, Embu MCB; EMGG, Embu MGG; EMMC, Embu EMMC; KMMC, Kitui MMC; KMPP, Kitui MPP; MMBB, Meru MBB; TMCB, Tharaka Nithi MCB; TMGG, Tharaka Nithi MGG; (**C**) cropping systems; (**D**) study locations.

**Figure 8:**
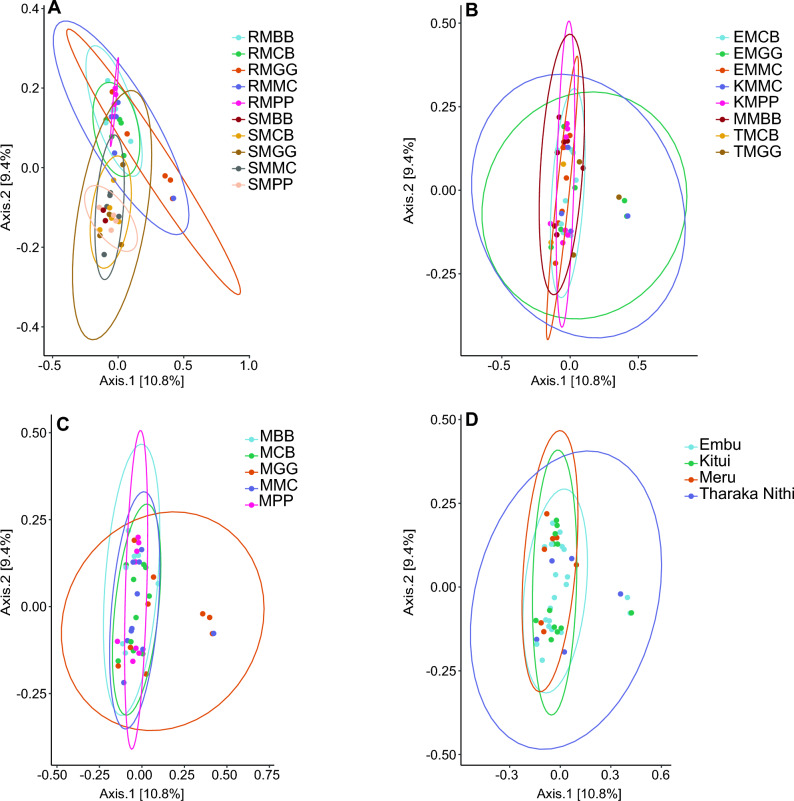
Beta diversity of bacterial species communities. (**A**) cropping systems and sample types; maize-root (R), and rhizospheric soil (S) samples; MPP, maize-pigeon pea; MBB, maize-black bean; MCB, maize-common bean; MGG, maize-green gram; and MMC, maize-monoculture cropping systems; (**B**) cropping systems and study locations; EMCB, Embu MCB; EMGG, Embu MGG; EMMC, Embu EMMC; KMMC, Kitui MMC; KMPP, Kitui MPP; MMBB, Meru MBB; TMCB, Tharaka Nithi MCB; TMGG, Tharaka Nithi MGG; (**C**) cropping systems; (**D**) study locations.

Visualization and quantification of β-diversity differences in fungal and bacterial communities revealed distinct clustering based on different cropping systems. There was a significant microbial variation between MLI and MMC cropping systems, sample types, and study locations. Fungal communities showed clear separation along axis 1, with a subtle clustering by MLI and MMC cropping systems, sample types, and study locations along axis 2 (Fig. 7). Similar clustering patterns were observed in maize-root and rhizospheric soil bacterial cropping systems communities (Fig. 8).

Each cropping system, sample type, and study location contributed to distinct fungal and bacterial species habitats in the maize-root and rhizospheric soil, harboring different microbiomes alongside shared ones between MLI and MMC habitats. These differences were confirmed by PERMANOVA, highlighting the marked fungal and bacterial distinctions between cropping systems, sample types, and study locations. Additionally, individual cropping systems (MBB, MPP, MCB, MGG, and MMC) exhibited varying abundant sequence variant species but without consistency between them. In fungal species, comparisons revealed a significant difference between cropping systems and sample types (PERMANOVA; *R*^*2*^ = 0.364, *P* < 0.001, *df* = 9, *F* = 1.476), cropping systems and study locations (*R*^2^ = 0.256, *P* < 0.001, *df* = 7, *F* = 1.476), and study location (*R*^2^ = 0.137, *P* < 0.001, *df* = 3, *F* = 1.800). However, there was no statistical difference between cropping systems (*R*^2^ = 0.104, *P* = 0.080, *df* = 4, *F* = 1.135) (Table S21). In bacterial species, there was a significant difference between cropping systems and sample types in bacterial species (*R*^2^ = 0.257, *P* < 0.001, *df* = 9, *F* = 1.311). However, there were no statistical differences between cropping systems (*R*^2^ = 0.097, *P* = 0.262, *df* = 4, *F* = 1.054), cropping systems and study locations (*R*^2^ = 0.151, *P* = 0.910, *df* = 7, *F* = 0.914) and study location (*R*^2^ = 0.055, *P* = 0.995, *df* = 3, *F* = 0.781) (Table S22).

**Figure 9:**
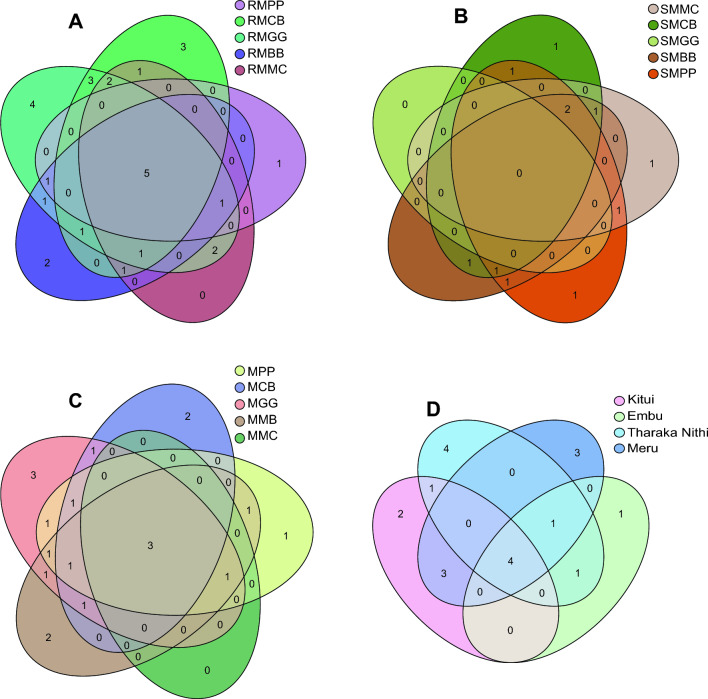
Venn diagram of fungal species shared between. (**A**) cropping systems and maize-root (R) samples type; MPP, maize-pigeon pea; MBB, maize-black bean; MCB, maize-common bean; MGG, maize-green gram; and MMC, maize-monoculture cropping systems; (**B**) cropping systems and rhizospheric soil (S) samples type; (**C**) cropping systems; (**D**) study locations.

Venn diagram demonstrated that the predominant and unique fungal species were found in 90% of the sampled ASVs, maintaining a steady 75% prevalence across MLI (MBB, MCB, MPP, and MGG) and MMC cropping systems. When comparing cropping systems and sample types interaction in maize-root (R), RMGG had four unique fungal species, RMCB had three, and RMBB had two, while RMPP had only one fungal species compared to RMMC. Similarly, regarding rhizospheric soil (S) samples, SMBB had 12 unique fungal species, while SMPP, SMCB, and SMMC had one unique fungal species. Five overlapping fungal species were shared between cropping systems and maize-root sample types (Fig. 9A and B). Based on cropping systems (MLI and MMC), MGG had three unique fungal species, MCB and MBB had two unique fungal species, while MPP had only one unique species compared to the MMC cropping systems. The MLI and MMC cropping systems shared three overlapping fungal species (Fig. 9C). In study locations, Tharaka Nithi had four different unique fungal species, Meru had three unique fungal species, Kitui had two unique species, and Embu had only one species (Fig. 9D). Four overlapping fungal species were shared across the study locations.

**Figure 10:**
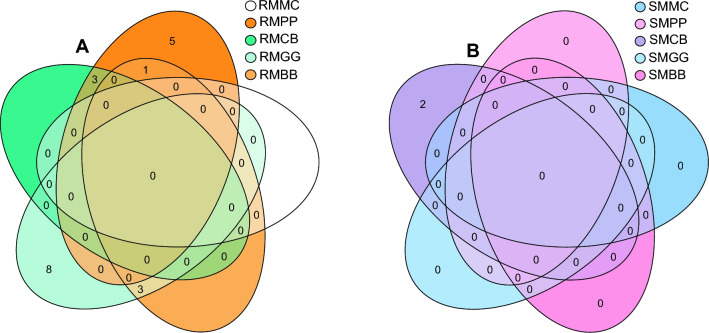
Venn diagram of bacterial species shared between. (**A**) cropping systems and maize-root (R) samples type; MPP, maize-pigeon pea; MBB, maize-black bean; MCB, maize-common bean; MGG, maize-green gram; and MMC, maize-monoculture cropping systems; (**B**) cropping systems and rhizospheric soil (S) samples type.

Interestingly, based on bacterial species, when comparing cropping systems and sample types interaction in maize-root, RMMC had 46 unique species, RMCB had 22, RMBB had 11, RMGG had eight, and RMPP had five bacterial species. However, regarding rhizospheric soil (S) samples, only SMMC had two unique bacterial species, while SMPP, SMCB, SMPP, and SMMC had no unique bacterial species (Fig. 10).

### Soil physico-chemical properties

The soil parameters in four maize edible-legume intercropping systems (MBB, MPP, MCB, and MGG) differed significantly from maize-monoculture (MMC) cropping systems. Notably, all soil physico-chemical parameters, except for S (sulphur), Fe (iron), Zn (zinc), Mn (manganese), P (phosphorus), K (potassium), EC, (electrical conductivity), sand, and Mg (magnesium), exhibited significant differences (ANOVA, *P* < 0.05; Table 2). It is worth mentioning that the lowest pH values were recorded in the MMC soil. In contrast, the soils derived from the different MLI systems exhibited higher pH, N (nitrogen), OC (organic carbon), EA (exchangeable acidity), Ca (calcium), and Cu (copper) values compared to that of MMC soils (Table 2).

**Table 2:** Soil physico-chemical properties of maize edible-legume intercropping and maize-monoculture cropping systems obtained from smallholder farmer fields.

Soil parameters	Maize edible-legume intercropping systems						
	MBB	MPP	MCB	MGG	MMC	*F* value (5,25)	*P* value
pH (H_2_O)	6.15 ± 0.19^b^	7.45 ± 0.19^c^	5.95 ± 0.19^ab^	6.53 ± 0.19^b^	5.19 ± 0.19^a^	19.697	** < 0.001**
EC (mmhos/cm)	0.11 ± 0.10^a^	0.37 ± 0.10^a^	0.07 ± 0.10^a^	0.30 ± 0.10^a^	0.06 ± 0.10^a^	2.042	> 0.119
P (mg/kg)	24.48 ± 15.72^a^	44.45 ± 15.72^a^	14.15 ± 15.72^a^	42.91 ± 15.72^a^	14.43 ± 15.72^a^	0.743	> 0.572
K (mg/kg)	436.38 ± 97.20^a^	569.64 ± 97.20^a^	264.46 ± 97.20^a^	427.73 ± 97.20^a^	186.88 ± 97.20^b^	1.924	> 0.138
Na (mg/kg)	44.63 ± 14.60^b^	114.51 ± 14.60^b^	43.72 ± 14.60^ab^	58.53 ± 14.60^ab^	51.41 ± 14.60^b^	4.112	** < 0.001**
Ca (mg/kg)	1791.10 ± 346.00^ab^	2876.61 ± 346.00^b^	1340.79 ± 346.00^a^	1410.05 ± 346.00^b^	889.18 ± 346.00^a^	4.706	** < 0.001**
Mg (mg/kg)	302.11 ± 76.10^a^	371.41 ± 76.10^a^	252.14 ± 76.10^a^	380.92 ± 76.10^a^	167.57 ± 76.10^a^	1.354	> 0.279
EA (meq/100 g)	0.43 ± 0.10^b^	*NA*	0.37 ± 0.10^ab^	*NA*	0.25 ± 0.10^ab^	3.894	** < 0.010**
Fe (mg/kg)	127.76 ± 17.00^a^	69.63 ± 17.00^a^	102.47 ± 17.00^a^	112.13 ± 17.00^a^	89.48 ± 17.00^a^	1.689	> 0.184
Mn (mg/kg)	233.11 ± 46.30^a^	169.02 ± 46.30^a^	221.37 ± 46.30^a^	193.40 ± 46.30^a^	244.82 ± 46.30^a^	0.444	> 0.775
Cu (mg/kg)	4.34 ± 1.15^a^	1.95 ± 1.15^a^	2.99 ± 1.15^a^	5.86 ± 1.15^a^	1.73 ± 1.15^a^	2.264	> 0.090
Zn (mg/kg)	7.09 ± 1.97^a^	3.57 ± 1.97^a^	7.74 ± 1.97^a^	5.64 ± 1.97^a^	6.31 ± 1.97^a^	0.666	> 0.623
S (mg/kg)	12.99 ± 8.02^a^	36.12 ± 8.02^a^	14.24 ± 8.02^a^	21.61 ± 8.02^a^	19.26 ± 8.02^a^	1.327	> 0.288
N (%)	0.29 ± 0.02^b^	0.16 ± 0.02^a^	0.16 ± 0.02^ab^	0.24 ± 0.02^ab^	0.20 ± 0.02^ab^	5.887	** < 0.001**
OC (%)	3.02 ± 0.27^b^	1.56 ± 0.27^a^	1.64 ± 0.27^a^	2.41 ± 0.27^ab^	2.06 ± 0.27a^b^	5.140	** < 0.001**
Clay (%)	28.74 ± 3.92^a^	21.67 ± 3.92^a^	34.16 ± 3.92^a^	34.55 ± 3.92^a^	37.90 ± 3.92^a^	2.627	** < 0.050**
Sand (%)	30.40 ± 7.05^a^	57.10 ± 7.05^a^	41.70 ± 7.05^a^	43.60 ± 7.05^a^	36.30 ± 7.05^a^	2.160	> 0.103
Silt (%)	40.82 ± 5.07^a^	21.25 ± 5.07^a^	24.16 ± 5.07^a^	30.81 ± 5.07^a^	25.83 ± 5.07^a^	2.160	** < 0.010**

MBB, maize-black bean; MPP, maize-pigeon pea; MCB, maize-common bean; MGG, maize-green gram; and MMC, maize-monoculture cropping systems soil; pH, potential of hydrogen; EC, electrical conductivity; mmhos/cm, millimhos per centimeter; P, phosphorus; mg/kg, milligrams per kilogram; K, potassium; Na, sodium; Ca, calcium; Mg, magnesium; Fe, iron; Mn, manganese; Cu, cupper; Zn, zinc; B, boron; Mo, molybdenum; S, sulphur; N, nitrogen; %, percentage; OC, organic carbon; *NA*, Negligible; EA, exchangeable acidity; meq/100 g, millequivalents per 100 g of soil. Different letter indicates significant difference among the different maize edible-legume intercropping systems and MMC soils (*P* < 0.05) according to Tukeys honest significance test (HSD), and significant effects are indicated in bold at *P* < 0.05.

**Figure 11:**
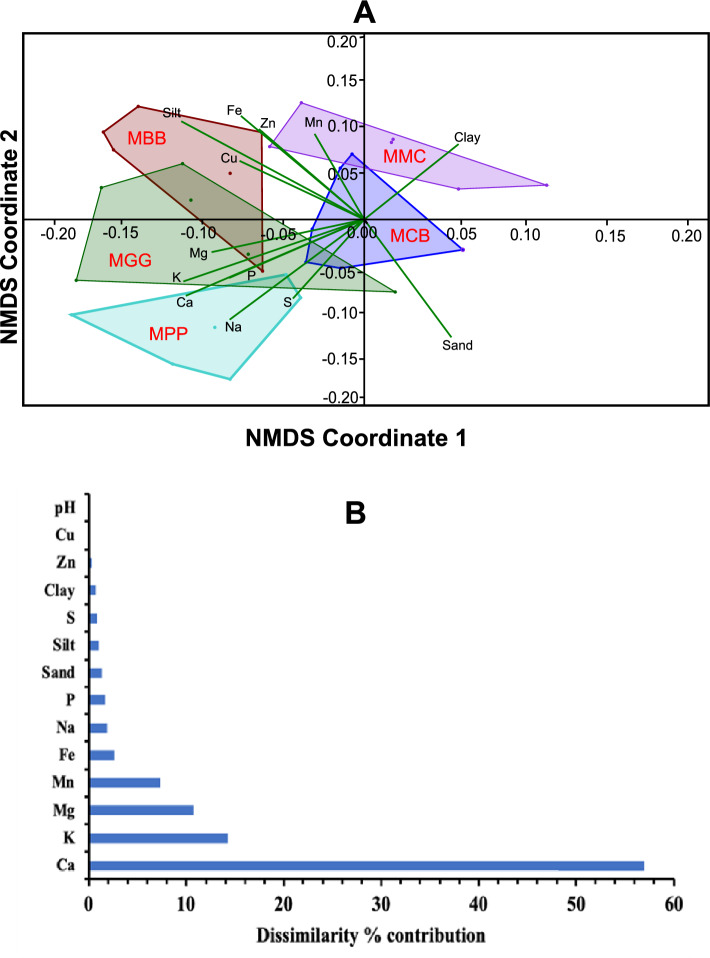
Impact of soil physico-chemical properties on different cropping systems. (**A**) non-metric multidimensional scaling (NMDS) plot, illustrates the clustering patterns among the soil physico-chemical properties in different cropping systems; (**B**) histogram illustrates the predominant percentage contribution of soil chemical properties based on their similarities. Maize-black bean, MBB; maize-green gram, MGG, maize-pigeon pea, MPP; maize-common bean, MCB, maize-monoculture cropping systems, MMC; Ca, calcium; Mn, manganese; Mg, magnesium; Fe, iron; K, potassium; P, phosphorus; S, sulphur; Zn, zinc; Cu, copper; Na, sodium; pH, potential of hydrogen; OC, organic carbon; and N, nitrogen.

We found significant differences between the soil physico-chemical parameters among the different MLI and MMC cropping systems soils. (One-way ANOSIM with a *P* < 0.0003, an *R* = 0.2909, and a *Stress value* = 0.1894; Fig. 11A). The following specific soil physico-chemical properties predominantly accounted for the variations and distinguishing properties between the MLI and MMC cropping systems: Ca (57.01%), K (14.22%), Mg (10.69%), Mn (7.31%), Fe (2.59%), Na (1.94%), P (1.65%), sand (1.33%), silt (1.04%), S (0.85%), clay (0.72%), Zn (0.29%), pH (0.09%), and OC (0.08%), collectively contributed to the observed dissimilarities (Fig. 11B).

## Discussion

Our research explored soil conditioning through maize edible-legume intercropping (MLI) systems, encompassing diverse aspects such as microbial abundance, diversity, and soil physico-chemical properties to favor soil health and sustainable agricultural production. The health of rhizosphere soil, influenced by factors such as crop diversification, among other cropping systems, plant species, and soil physico-chemical parameters, is crucial^4,31,43^.

In comparison to maize-monoculture cropping (MMC) systems, MLI systems were dominated by beneficial belowground fungi genera and species associated with improved soil fertility. Significant endophytic fungi identified in the MLI systems were *Bionectria solani*, *Sarocladium zeae*, and *Atractiella rhizophila*. Previous studies have attributed *Binonectria solani* to suppressing *Botryosphaeria corticola*, which causes stem cankers, vascular necrosis, and die-back in healthy cork oak trees^44^. Also, maize biocontrol experimental studies have shown the efficacy of *Sarocladium zeae* in establishing symptomless kernel associations and secondary metabolites production of pyrrocidine A and B, preventing kernel-rotting caused by *Fusarium graminearum*, *Fusarium verticillioides*, and mycotoxin-producing fungi (*Aspergillus flavus*)^45,46^. Similarly, *Atractiella rhizophila* asymptomatically colonizes plant roots of orchids (*Orchidaceae*), soybean, maize, and rice, subtly facilitating plant growth and photosynthesis^47^.

In the MLI systems, other endophytic fungi include *Alternaria angustiovoidea* that suppresses *Ralstonia solani* (which causes rice sheath blight)^48^; *Epicoccum dendrobii*, known for inhibiting anthracnose lesion development caused by pathogens like *Colletotrichum gloeosporioides*^49^; *Acremonium persicinum*, associated with the control of black leaf spots in coconut trees caused by *Camarotella torrendiella*, and *Camarotella acrocomiae*^50^. *Condenascus tortuosus* and *Fusarium algeriense* are entomopathogenic fungi against *Helicoverpa armigera* (Hübner). The presence of beneficial fungal species in MLI systems (both in rhizospheric soil and maize-root) in smallholder farmer fields enhances plant defence mechanisms of plant against pathogens, and mycotoxin-producing fungi, contributing to food safety and higher crop yields. In contrast, the MMC system had many harmful pathogenic fungi species, such as *Alternaria argyroxiphii*, *Stagonosporopsis trachelii*, and *Fusarium falciforme*. *Alternaria argyroxiphii* and *Stagonosporopsis trachelii,* cause leaf spots on *Khaya senegalensis* (African mahogany trees)^51^; and *Scrophularia ningpoensis*^52,53^. Several studies have shown that *Fusarium falciforme* causes root rot in *Glycine max* and *Phaseolus lunatus*, which are associated with *Fusarium* wilt of *Cannabis sativa* and bud rot of *Agave tequilana*^54,55^.

Long-term monocropping of maize has been reported to reduce key soil nutritive parameters such as total carbon, total nitrogen, available phosphorus, exchangeable calcium, and potassium^56^. However, intercropping with legumes contributes to the fixation of atmospheric nitrogen and adds crop residues that are rich in proteins, calcium, and other nutrients to the soil^57^. The diversity of beneficial microbes in the soil contributes to the effective degradation of crop residues and ensures enhanced availability of nutrients^58^. In this study, the distinct bacterial communities in the MLI system rhizospheric soil suggest crop diversification influences belowground microbiomes both in the rhizospheric soil and crop roots. Of particular significance is *Rhizobium daejeonense*, which has environmental and agricultural impact due to its symbiotic relationship with legume plants, production of indole-3-pyruvic acid (IAA), phosphate solubilization, and carbamazepine degradation^59,60^. The prevalence of *Bacillus aryabhattai* in MLI systems has been shown to facilitate zinc assimilation, improving plant growth through biofortification^61^. Notably, the relative abundance of *Amycolatopsis mediterranei* in MLI systems is valuable for antibiotic production, specifically rifamycin, which is effective against pathogenic mycobacteria^62,63^. *Bacillus pseudofirmus* and *Bacillus megaterium* present in MLI systems are associated with solubilizing soil nutrients (P and K) and inhibiting brown root rot disease caused by *Fomes lamaoensis*^64,65^. Lee et al.^66^ and Herrera et al.^67^ have shown *Dyella marensis*, which was more relatively abundant in MLI system rhizospheric soil, is effective in the degradation of biphenyl compounds and can produce siderophores and biosurfactants vital in promoting plant and soil health.

Field co-inoculation trials with endophytic nitrogen-fixing bacteria in MLI systems, such as *Bradyrhizobium elkanii*, *Bradyrhizobium liaoningense*, *Bradyrhizobium yuanmingense*, and *Sinorhizobium fredii*, have been attributed to the promotion of plant growth by enhancing resource uptake, nitrogen fixation, nodulation, chlorophyll synthesis, and contribute to soil fertility^68^. These ecological services can contribute to improving soil fertility and decreasing reliance on synthetic chemical fertilizer inputs. A combination of such beneficial microbial species has been shown to promote the production of phytohormones like IAA, which promotes plant cell growth. Additionally, these beneficial microorganisms have a greater potential as effective biofertilizers, particularly for boosting the yield of crucial horticultural crops^69–71^. In addition, *Catenulispora acidiphila*, a novel actinomycetes, was relatively abundant in MLI systems smallholder farms, associated with debris decomposition, secondary metabolites production, carbon cycle, and the production of antimicrobial properties^72,73^.

Hence, the identified dominant microbes within the MLI systems, comprising both fungi and bacteria, could have played crucial roles in improving soil fertility, production of siderophores, carbon sequestering, nutrient cycling, synthesis of phytohormones, and protection of plants when compared to that of MMC cropping systems^4,7,10,21,74,75^. This trend aligns with similar studies on cereal and legume intercropping systems, such as wheat-soybean, cowpea-melon, peanut-maize, and millet-mung bean^20,21,28,43,76^, and push–pull smallholder farmer plots^4^, emphasizing the primary objective of diversification to improve food security, soil fertility and reduce cardon sequestration^28,74^. This also aligns with our study results on the positive impact of different MLI systems on soil pH, N, OC, Ca, Na, and P levels in comparison to MMC system. In contrast, MMC system smallholder farms exhibited an aggregate pH below 5.5, reinforcing the validity and consistency of poorly performing maize crops in these systems^23,43,77^. Remarkably, MLI systems displayed higher pH values ranging between 5.95 and 7.45, suggesting a more favorable soil environment for microbial activities that support nutrient cycle, phytohormone production, and protect plant growth. The soil pH and micronutrients are reliable indicators for soil health, as they are crucial for crop resilience^74,78^. These findings align with previous research indicating a positive relationship between the composition of belowground fungal and bacterial communities in the rhizosphere and soil physico-chemical characteristics^4,21,23,79^. These findings underscore the critical role of MLI systems in sustaining agroecosystem functions, offering extremely significant agricultural benefits, and potentially contributing to increased maize yields. This favorable environment enhances defense mechanisms and provides other important ecological services that promote plant production^75,80–82^. These beneficial belowground bacteria align with higher maize yield and soil fertility observed in MLI fields compared to the MMC farms^7^. These results substantiate the prediction that crop diversification profoundly influences soil biological, physical, and chemical properties.

## Conclusion

We have demonstrated that crop mixtures play a transformative role in restructuring rhizospheric soil and maize-root microbial communities, thereby influencing critical soil physico-chemical properties within smallholder farm fields. This restructuring mitigates risks associated with monoculture cropping system. It promotes the proliferation of the beneficial microorganisms associated with important ecological services, including enhancing soil fertility, organic matter decomposition, carbon sequestration, plant protection, and ultimately contribute to food safety. Enriching these vital microbial communities expands the range of agroecosystem services offered by the cropping systems, fostering resilience and augmenting functional diversity. Subsequent research should delve into the effects of MLI systems on different crops, identifying and harnessing influential microbial communities. Additionally, there is a need to elucidate the underlying mechanisms, including the role of plant metabolites and volatile organic compounds through root microbial exudates. Further investigations should pinpoint specific microorganisms affected by MLI systems, elucidate their roles in aboveground interactions, and assess their impact on soil properties within smallholder fields where this cropping system is practiced. This study lays a foundation for incorporating microbial diversity management approaches into smart farming systems, and highlights how different crop diversifications influence microbiota in both soil and maize-root.

### Supplementary Information

Supplementary Information.

## Data Availability

The unprocessed sequencing datasets generated during the current study have been deposited in GenBank, NCBI under BioProject PRJNA1056154. The 16S (V3-V4, bacteria) and ITS (ITS1-ITS2, fungi) metagenome data were registered as Biosamples SAMN39455314–SAMN39455397 and the sequences assigned SRA accessions SRR27606564–SRR27606647.
