# Supplementary material for: Maize edible-legumes intercropping systems for enhancing agrobiodiversity and belowground ecosystem services
Source: Sci Rep. 2024 Jun 21;14:14355. doi: 10.1038/s41598-024-64138-w (PMC11192945; doi:10.1038/s41598-024-64138-w)
Supplement: Supplementary file 1 — Supplementary Information. [file 41598_2024_64138_MOESM1_ESM.pdf]

Maize Edible-Legumes Intercropping Systems for Enhancing Agrobiodiversity and Belowground Ecosystem Services

Abdul A. Jalloh[1,2], Daniel Munyao Mutyambai[1,3]*, Abdullahi Ahmed Yusuf[2,4], Sevgan Subramanian[1], Fathiya Khamis[1]

[1]*International Centre of Insect Physiology and Ecology, P.O. Box 30772-00100 Nairobi, Kenya.*
[2]*Department of Zoology and Entomology, University of Pretoria, Pretoria, Private Bag x20 Hatfield, South Africa.*
[3]*Department of Life Sciences, South Eastern Kenya University, P.O Box 170-90200, Kitui, Kenya.*
[4]*Forestry and Agricultural Biotechnology Institute, University of Pretoria, Pretoria, Private Bag x20 Hatfield, South Africa.*
***Correspondence:** *dmutyambai@icipe.org*

Supplementary results

Abundance and taxonomic profiling of fungal and bacterial communities in rhizospheric soil and maize-root from different cropping systems

The effects of fungal community genera were influenced differently by the interaction between MLI and MMC cropping systems based on study locations, cropping systems, and sample types (R for maize-root samples and S for rhizospheric soil samples). The most abundant fungal genera in Embu RMMC, Meru RMBB, and Meru SMBB were *Setophoma* (74.00%, 53.60%, and 23.40%), respectively. *Cladosporium* was more enriched in Embu RMCB, Tharaka Nithi RMCB, Embu SMGG, and Kitui (42.10%, 39.70%, 22.00%, and 9.50%), while *Preussia* (57.40%) were highly abundant in Embu SMMC. *Neocosmopora* (48.70%) were abundant in Kitui RMMC compared to the other study locations, sample types, and cropping systems. *Hannaella* (15.10%) showed higher enrichment in Kitui RMMC. *Gibberrella* (15.10%) was more enriched in Embu RMGG. Fusarium (46.00%) were more abundant in Kitui RMPP, while *Epicoccum* (54.90%) were highly abundant in Tharaka Nithi SMCB when compared to the other study locations, different sample types and cropping systems **(Fig. S1C; Table S3)**.

The interaction between MLI and MMC cropping systems had varying effects on the impact of bacterial community genera in cropping systems based on study locations, cropping systems, and sample types. The most abundant bacterial genera in Tharaka Nithi SMCB, Embu SMMC, Embu SMCB, Meru SMBB, Kitui SMMC, Embu RMCB, and Kitui SMPP were *Bacillus* (55.30%, 52.00%, 50.00%, 45.60%, 41.30%, 28.80%, and 2640%), respectively. At the same time, *Bradyrhizobium* (76.50%, 71.60%, and 60.00%) was more abundant in Embu RMGG, Tharaka Nithi RMGG, and Kitui RMMC, respectively. *Ralstonia* (37.20%, and 31.40%) showed higher enrichment in Meru RMBB and Embu RMMC. Kitui RMPP showed a higher abundance of *Mitsuaria* genus than the other study locations, with different sample types and cropping systems. *Allorhizobium-Neorhizobium-Pararhizobium-Rhizobium* were more abundant in Tharaka Nithi RMBB **(Fig. S2C; Table S9)**.

Supplementary figures

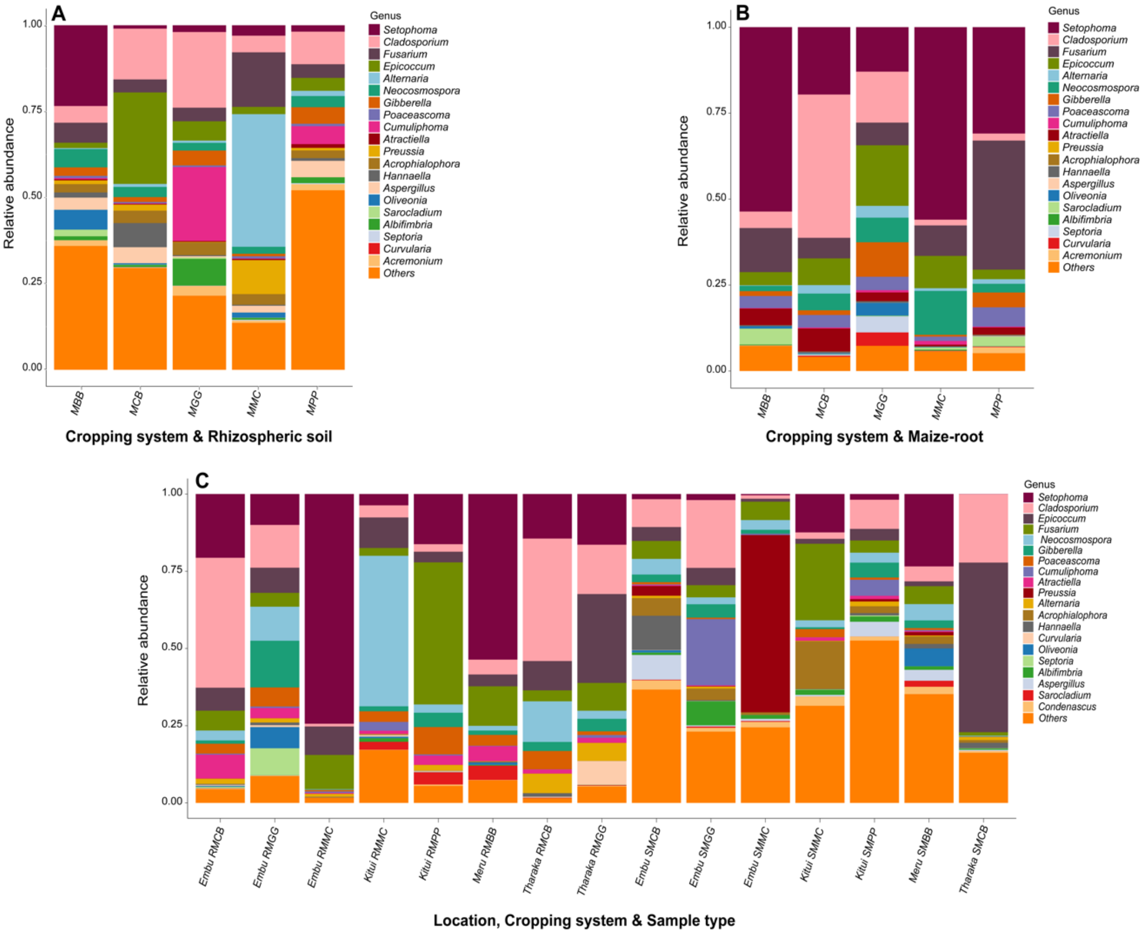

Figure S1: Relative abundance of fungal genera. **(A)** cropping systems and rhizospheric soil samples; R, maize-root sample; S, rhizospheric soil sample; MPP, maize-pigeon pea; MBB, maize-black bean; MCB, maize-common bean; MGG, maize-green gram; and MMC, maize-monoculture cropping systems; **(B)** cropping systems and maize-root samples; **(C)** study locations, cropping systems, and sample types. Fungal genera with relative abundance lower than 1% were grouped as others.

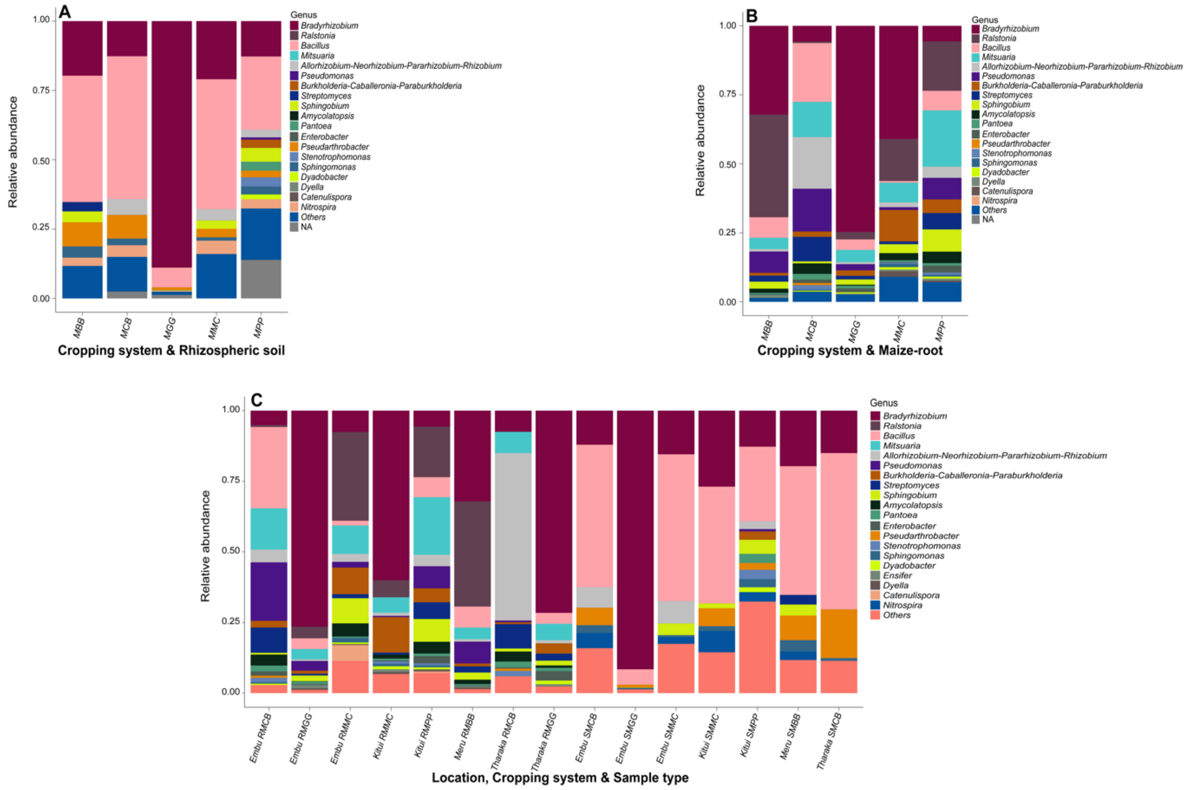

Figure S2: Relative abundance of bacterial genera. **(A)** cropping systems and rhizospheric soil samples; R, maize-root sample; S, rhizospheric soil sample; MPP, maize-pigeon pea; MBB, maize-black bean; MCB, maize-common bean; MGG, maize-green gram; and MMC, maize-monoculture cropping systems; **(B)** cropping systems and maize-root samples; **(C)** study locations, sample types, and cropping systems. Bacterial genera with relative abundance lower than 1% were grouped as others.

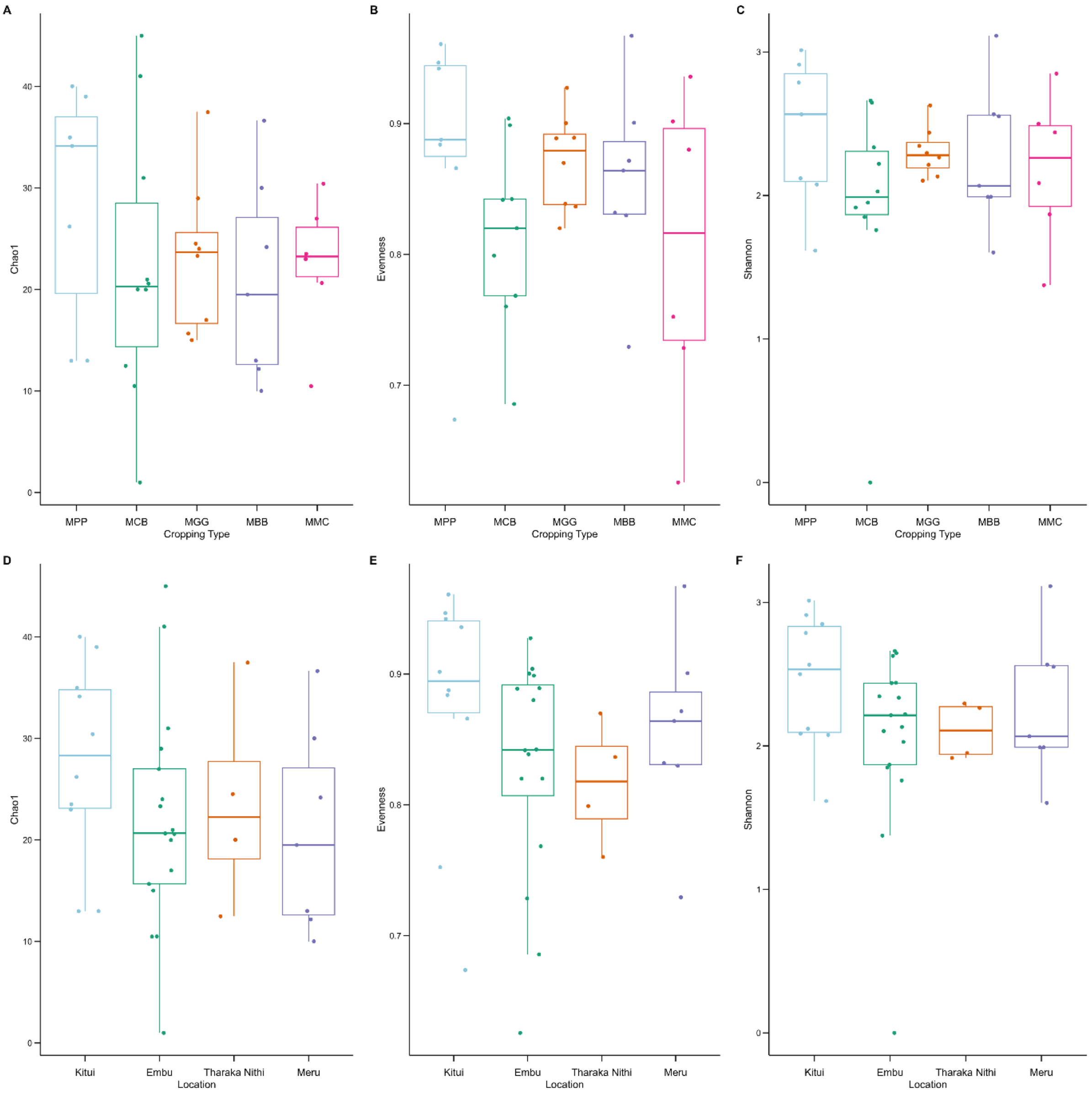

Figure S3: Alpha diversity of fungal communities. **(A, B, C)** cropping systems; MPP, maize-pigeon pea; MBB, maize-black bean; MCB, maize-common bean; MGG, maize-green gram; and MMC, maize-monoculture cropping systems; **(D, E, F)** study locations.

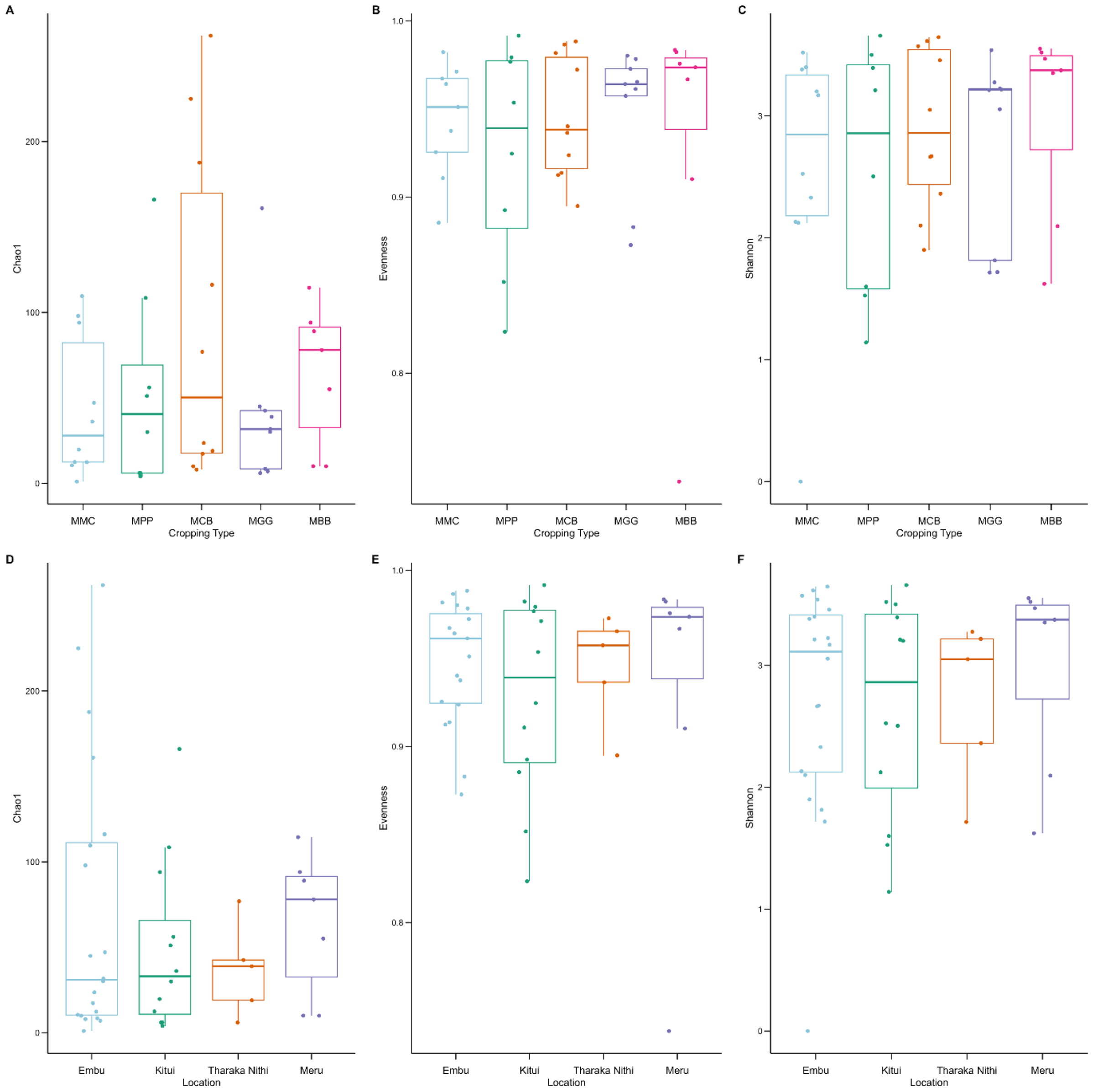

Figure S4: Alpha diversity of bacterial communities. **(A, B, C)** cropping systems; MPP, maize-pigeon pea; MBB, maize-black bean; MCB, maize-common bean; MGG, maize-green gram; and MMC, maize-monoculture cropping systems; **(D, E, F)** study locations.

Supplementary tables

Table S1: Relative abundance (%) of fungal genera communities in different cropping systems and rhizospheric soil types. Fungal genera with relative abundances < 1 were grouped as 'other'.

S/No	Genus	Percentage relative abundance				
		**MBB**	**MCB**	**MGG**	**MMC**	**MPP**
1	*Acremonium*	1.70	0.20	2.80	0.70	1.80
2	*Acrophialophora*	2.40	3.60	3.80	3.10	2.20
3	*Albifimbria*	1.10	0.60	7.80	0.50	1.70
4	*Alternaria*	0.30	0.90	0.70	38.50	1.50
5	*Aspergillus*	3.50	4.70	0.40	2.00	4.80

6	*Atractiella*	0.30	0.20	0.30	0.40	1.10
7	*Cladosporium*	4.90	14.80	22.00	4.80	9.50
8	*Cumuliphoma*	0.40	0.10	21.50	0.10	5.20
9	*Curvularia*	0.00	0.10	0.00	0.00	0.00
10	*Epicoccum*	1.60	26.60	5.60	2.10	3.70
11	*Fusarium*	5.80	3.80	3.90	15.80	4.00
12	*Gibberella*	2.50	1.40	4.40	0.70	4.80
13	*Hannaella*	1.50	7.00	0.30	0.30	0.80
14	*Neocosmospora*	5.30	2.90	2.20	2.00	3.20
15	*Oliveonia*	5.80	0.40	0.00	1.50	0.00
16	Others	35.90	29.50	21.50	13.60	52.10
17	*Poaceascoma*	0.60	0.40	0.50	0.60	0.70
18	*Preussia*	1.10	1.80	0.10	9.80	0.70
19	*Sarocladium*	1.90	0.10	0.40	0.10	0.00
20	*Septoria*	0.00	0.10	0.10	0.20	0.30
21	*Setophoma*	23.40	1.00	1.90	3.00	1.80
	**Total**	**100.00**	**100.00**	**100.00**	**100.00**	**100.00**

MPP, maize-pigeon pea; MBB, maize-black bean; MCB, maize-common bean; MGG, maize-green gram; and MMC, maize-monoculture cropping systems.

Table S2: Relative abundance (%) of fungal genera communities in different cropping systems and maize-root types. Fungal genera with relative abundances < 1 were grouped as 'other'.

**S/No**	**Genus**	**Percentage relative abundance**				
		**MBB**	**MCB**	**MGG**	**MMC**	**MPP**
1	*Acremonium*	0.20	0.00	0.00	0.10	1.80
2	*Acrophialophora*	0.00	0.00	0.00	0.00	0.10
3	*Albifimbria*	0.20	0.00	0.10	0.30	0.10
4	*Alternaria*	0.20	2.50	3.40	0.80	1.40
5	*Aspergillus*	0.00	0.10	0.00	0.00	0.20
6	*Atractiella*	4.80	6.70	2.60	0.50	2.10
7	*Cladosporium*	4.80	41.70	14.80	1.70	2.10
8	*Cumuliphoma*	0.20	0.30	0.70	1.10	0.20
9	*Curvularia*	0.00	0.30	3.90	0.10	0.20
10	*Epicoccum*	3.80	7.80	17.60	9.40	2.80
11	*Fusarium*	12.70	6.00	6.60	8.80	37.50
12	*Gibberella*	1.40	1.40	10.00	0.50	4.30
13	*Hannaella*	0.30	0.40	0.50	0.20	0.30
14	*Neocosmospora*	1.50	4.80	7.10	12.70	2.50
15	*Oliveonia*	0.70	0.30	3.70	0.10	0.00
16	Others	7.30	4.10	7.30	5.70	5.20
17	*Poaceascoma*	3.50	3.60	3.90	1.20	5.60
18	*Preussia*	0.10	0.00	0.00	0.00	0.00
19	*Sarocladium*	4.60	0.10	0.10	0.70	2.70

20	*Septoria*	0.00	0.30	4.70	0.00	0.00
21	*Setophoma*	53.60	19.60	12.90	56.00	30.90
	**Total**	**100.00**	**100.00**	**100.00**	**100.00**	**100.00**

MPP, maize-pigeon pea; MBB, maize-black bean; MCB, maize-common bean; MGG, maize-green gram; and MMC, maize-monoculture cropping systems.

Table S3: Relative abundance (%) of fungal genera communities among study locations, cropping systems, and sample types. Fungal genera with relative abundances < 1 were grouped as 'other'.

S/No	Genus	Percentage relative abundance														
		**Embu RMCB**	**Embu RMGG**	**Embu RMMC**	**Kitui RMMC**	**Kitui RMPP**	**Meru RMBB**	**Tharaka Nithi RMCB**	**Tharaka Nithi RMGG**	**Embu SMCB**	**Embu SMGG**	**Embu SMMC**	**Kitui SMMC**	**Kitui SMPP**	**Meru SMBB**	**Tharaka Nithi SMCB**
1	*Acrophialophora*	0.00	0.00	0.00	0.00	0.10	0.00	0.00	0.00	5.70	3.80	0.80	15.40	2.20	2.40	0.90
2	*Albifimbria*	0.00	0.10	0.00	1.20	0.10	0.20	0.00	0.10	0.70	7.80	1.20	1.70	1.70	1.10	0.40
3	*Alternaria*	1.70	1.40	1.00	0.20	1.80	0.20	6.40	5.90	0.80	0.70	0.00	0.10	1.50	0.30	1.00
4	*Aspergillus*	0.10	0.10	0.00	0.00	0.10	0.00	0.00	0.00	8.00	0.40	0.70	0.50	4.80	3.50	0.40
5	*Atractiella*	7.80	3.30	0.30	1.10	3.00	4.80	1.30	1.60	0.30	0.30	0.00	1.10	1.10	0.30	0.00
6	*Cladosporium*	42.10	13.90	0.90	3.90	2.40	4.80	39.70	16.00	9.00	22.00	1.10	2.10	9.50	4.90	22.10
7	*Condenascus*	0.30	0.20	0.00	0.10	0.50	0.10	0.00	0.30	3.00	1.30	1.80	3.00	1.30	2.40	0.60
8	*Cumuliphoma*	0.30	0.50	0.50	2.80	0.40	0.20	0.10	0.90	0.20	21.50	0.50	0.10	5.20	0.40	0.00
9	*Curvularia*	0.30	0.60	0.00	0.50	0.30	0.00	0.30	7.70	0.10	0.00	0.00	0.00	0.00	0.00	0.00
10	*Epicoccum*	7.40	8.10	9.20	9.90	3.50	3.80	9.50	28.80	4.50	5.60	0.90	1.60	3.70	1.60	54.90
11	*Fusarium*	6.40	4.50	11.10	2.50	46.00	12.80	3.60	9.00	5.80	3.90	5.90	24.80	4.00	5.80	1.10
12	*Gibberella*	1.10	15.10	0.20	1.60	4.70	1.40	2.90	4.10	2.50	4.40	1.30	0.60	4.80	2.50	0.10
13	*Hannaella*	0.30	0.80	0.20	0.10	0.00	0.30	1.10	0.00	11.10	0.30	0.00	0.10	0.80	1.50	1.80
14	*Neocosmospora*	3.20	11.00	0.10	48.70	2.70	1.50	13.20	2.60	5.00	2.20	3.10	2.20	3.20	5.30	0.20
15	*Oliveonia*	0.30	6.90	0.00	0.50	0.00	0.70	0.00	0.00	0.70	0.00	0.00	0.00	0.00	5.80	0.00
16	Others	4.40	8.70	1.70	17.10	5.50	7.30	1.50	5.30	36.70	23.10	24.40	31.40	52.50	35.20	16.20
17	*Poaceascoma*	3.20	6.20	0.50	3.40	8.80	3.50	5.90	1.20	0.70	0.50	0.00	2.60	0.70	0.60	0.00
18	*Preussia*	0.00	0.00	0.00	0.00	0.00	0.00	0.00	0.00	3.10	0.10	57.40	0.00	0.70	1.10	0.00
19	*Sarocladium*	0.10	0.00	0.00	2.60	4.00	4.60	0.10	0.20	0.20	0.40	0.20	0.00	0.00	1.90	0.00
20	*Septoria*	0.40	8.70	0.00	0.00	0.00	0.00	0.00	0.00	0.00	0.10	0.00	0.10	0.30	0.00	0.10
21	*Setophoma*	20.60	10.00	74.40	3.70	16.20	53.60	14.40	16.40	1.70	1.90	0.40	12.40	1.80	23.40	0.10
	**Total**	**100.00**	**100.00**	**100.00**	**100.00**	**100.00**	**100.00**	**100.00**	**100.00**	**100.00**	**100.00**	**100.00**	**100.00**	**100.00**	**100.00**	**100.00**

R for maize-root samples and S for rhizospheric soil samples; MPP, maize-pigeon pea; MBB, maize-black bean; MCB, maize-common bean; MGG, maize-green gram; and MMC, maize-monoculture cropping systems.

Table S4: Relative abundance (%) of fungal species communities in different cropping systems and rhizospheric soil types. Fungal species with relative abundances < 1 were grouped as 'other'.

S/No	Species	Percentage relative abundance				
		MBB	MCB	MGG	MMC	MPP
1	*Acremonium persicinum*	0.20	0.10	2.80	0.40	0.10
2	*Acrophialophora hechuanensis*	2.10	1.10	1.20	1.00	1.00
3	*Acrophialophora levis*	0.40	2.40	2.40	2.10	1.20
4	*Albifimbria verrucaria*	1.10	0.60	7.80	0.50	1.70
5	*Alternaria angustiovoidea*	0.30	0.90	0.60	23.00	0.80
6	*Alternaria argyroxiphii*	0.00	0.00	0.10	15.60	0.80
7	*Aspergillus pseudodeflectus*	0.90	0.30	0.20	1.50	0.20
8	*Atractiella rhizophila*	0.30	0.20	0.30	0.40	1.10
9	*Bionectria solani*	1.80	1.00	0.10	0.20	1.60
10	*Cladosporium delicatulum*	4.90	14.70	21.40	4.70	8.10
11	*Condenascus tortuosus*	2.40	2.00	1.30	0.90	1.30
12	*Cumuliphoma indica*	0.00	0.00	6.90	0.00	0.00
13	*Cumuliphoma omnivirens*	0.40	0.10	14.60	0.10	5.20
14	*Curvularia caricae-papayae*	0.00	0.10	0.00	0.00	0.00
15	*Cutaneotrichosporon debeurmannianum*	1.70	0.60	0.50	0.30	3.50
16	*Dictyosporium heptasporum*	0.60	1.90	0.20	0.30	0.00
17	*Ectophoma multirostrata*	6.00	0.80	0.90	0.00	0.00
18	*Edenia gomezpompae*	0.00	0.30	0.00	0.10	0.60
19	*Epicoccum dendrobii*	0.00	5.90	0.40	1.30	0.00
20	*Epicoccum thailandicum*	1.60	20.50	4.70	0.60	3.20
21	*Fusarium acutatum*	2.90	0.60	1.50	10.30	1.50
22	*Fusarium algeriense*	0.60	0.40	0.20	0.20	0.00
23	*Fusarium kyushuense*	1.50	1.60	1.40	5.00	1.90
24	*Fusarium ornamentatum*	0.50	0.00	0.20	0.10	0.10
25	*Gibberella intricans*	2.50	1.40	4.40	0.70	4.80
26	*Hannaella oryzae*	0.40	5.20	0.20	0.10	0.70
27	*Lectera longa*	1.10	1.30	0.90	0.00	0.20
28	*Memnoniella longistipitata*	0.00	2.60	2.20	0.00	0.30
29	*Neocosmospora falciformis*	5.30	2.90	2.20	2.00	3.20
30	*Neopyrenochaeta spp.*	0.00	0.30	2.30	0.70	2.40
31	*Oliveonia pauxilla*	5.80	0.40	0.00	1.50	0.00
32	Others	27.00	23.30	10.00	8.90	41.40
33	*Papiliotrema laurentii*	0.40	0.60	0.40	1.70	1.10
34	*Penicillium hetheringtonii*	0.10	0.20	3.00	0.80	4.70
35	*Poaceascoma spp.*	0.60	0.40	0.50	0.60	0.70
36	*Preussia flanaganii*	1.10	1.70	0.10	9.40	0.40
37	*Sarocladium zeae*	1.90	0.00	0.20	0.00	0.00

38	*Septoria cretae*	0.00	0.10	0.10	0.20	0.30
39	*Setophoma terrestris*	23.40	1.00	1.90	3.00	1.80
40	*Stagonosporopsis trachelii*	0.00	0.30	2.00	0.00	0.70
41	*Westerdykella cylindrica*	0.30	2.00	0.00	1.50	3.40
	**Total**	**100.00**	**100.00**	**100.00**	**100.00**	**100.00**

MPP, maize-pigeon pea; MBB, maize-black bean; MCB, maize-common bean; MGG, maize-green gram; and MMC, maize-monoculture cropping systems.

Table S5: Relative abundance (%) of fungal species communities in different cropping systems and maize-root types. Fungal species with relative abundances < 1 were grouped as 'other'.

S/No	Species	Percentage relative abundance				
		MBB	MCB	MGG	MMC	MPP
1	*Acremonium persicinum*	0.00	0.00	0.00	0.00	1.80
2	*Acrophialophora hechuanensis*	0.00	0.00	0.00	0.00	0.00
3	*Acrophialophora levis*	0.00	0.00	0.00	0.00	0.00
4	*Albifimbria verrucaria*	0.20	0.00	0.10	0.30	0.10
5	*Alternaria angustiovoidea*	0.20	2.30	3.40	0.60	1.10
6	*Alternaria argyroxiphii*	0.00	0.20	0.00	0.20	0.00
7	*Aspergillus pseudodeflectus*	0.00	0.00	0.00	0.00	0.20
8	*Atractiella rhizophila*	4.80	6.70	2.60	0.50	2.10
9	*Bionectria solani*	0.10	0.10	0.00	0.00	0.00
10	*Cladosporium delicatulum*	4.80	41.30	14.40	1.70	2.10
11	*Condenascus tortuosus*	0.10	0.20	0.20	0.00	0.30
12	*Cumuliphoma indica*	0.00	0.00	0.00	0.00	0.00
13	*Cumuliphoma omnivirens*	0.20	0.30	0.70	1.10	0.20
14	*Curvularia caricae-papayae*	0.00	0.30	3.60	0.00	0.20
15	*Cutaneotrichosporon debeurmannianum*	0.20	0.30	0.30	0.30	0.20
16	*Dictyosporium heptasporum*	0.00	0.00	0.00	1.20	0.10
17	*Ectophoma multirostrata*	0.00	0.00	1.30	0.00	0.00
18	*Edenia gomezpompae*	0.20	0.20	0.20	0.40	1.00
19	*Epicoccum dendrobii*	0.90	0.40	1.80	1.80	0.00
20	*Epicoccum thailandicum*	2.80	7.30	15.20	7.40	2.70
21	*Fusarium acutatum*	11.80	1.50	3.00	8.10	22.80
22	*Fusarium algeriense*	0.80	1.70	0.10	0.60	1.80
23	*Fusarium kyushuense*	0.20	1.10	0.60	0.10	12.40
24	*Fusarium ornamentatum*	0.00	1.60	2.40	0.10	0.20
25	*Gibberella intricans*	1.20	1.40	10.00	0.50	4.30
26	*Hannaella oryzae*	0.00	0.10	0.10	0.00	0.10
27	*Lectera longa*	0.00	0.00	0.10	0.00	0.00
28	*Memnoniella longistipitata*	0.00	0.00	0.00	0.00	0.00
29	*Neocosmospora falciformis*	1.50	4.80	7.10	12.70	2.50
30	*Neopyrenochaeta spp.*	0.00	0.00	0.20	0.10	0.00

31	*Oliveonia pauxilla*	0.70	0.30	3.70	0.10	0.00
32	Others	7.30	4.00	6.10	4.80	5.00
33	*Papiliotrema laurentii*	0.00	0.00	0.00	0.10	0.10
34	*Penicillium hetheringtonii*	0.20	0.10	0.60	0.00	0.00
35	*Poaceascoma spp.*	3.50	3.60	3.90	1.20	5.60
36	*Preussia flanaganii*	0.00	0.00	0.00	0.00	0.00
37	*Sarocladium zeae*	4.60	0.10	0.10	0.00	1.90
38	*Septoria cretae*	0.00	0.30	4.70	0.00	0.00
39	*Setophoma terrestris*	53.60	19.60	12.90	56.00	30.90
40	*Stagonosporopsis trachelii*	0.00	0.20	0.60	0.00	0.20
41	*Westerdykella cylindrica*	0.00	0.00	0.00	0.00	0.00
	**Total**	**100.00**	**100.00**	**100.00**	**100.00**	**100.00**

MPP, maize-pigeon pea; MBB, maize-black bean; MCB, maize-common bean; MGG, maize-green gram; and MMC, maize-monoculture cropping systems.

Table S6: Relative abundance (%) of fungal species communities in study locations, cropping systems, and sample types. Fungal species with relative abundances < 1 were grouped as 'other'.

S/No	Species	Percentage relative abundance														
		**Embu RMCB**	**Embu RMGG**	**Embu RMMC**	**Kitui RMMC**	**Kitui RMPP**	**Meru RMBB**	**Tharaka Nithi RMCB**	**Tharaka Nithi RMGG**	**Embu SMCB**	**Embu SMGG**	**Embu SMMC**	**Kitui SMMC**	**Kitui SMPP**	**Meru SMBB**	**Tharaka Nithi SMCB**
1	*Acremonium persicinum*	0.00	0.00	0.00	0.00	0.00	0.00	0.00	0.00	0.10	2.80	0.20	1.60	0.10	0.20	0.10
2	*Acrophialophora hechuanensis*	0.00	0.00	0.00	0.00	0.00	0.00	0.00	0.00	1.70	1.20	0.80	4.20	1.00	2.10	0.40
3	*Acrophialophora levis*	0.00	0.00	0.00	0.00	0.10	0.00	0.00	0.00	3.80	2.40	0.00	11.20	1.20	0.40	0.50
4	*Albifimbria verrucaria*	0.00	0.10	0.00	1.20	0.10	0.20	0.00	0.10	0.70	7.80	1.20	1.70	1.70	1.10	0.40
5	*Alternaria angustiovoidea*	1.60	1.40	0.70	0.20	1.80	0.20	5.70	5.90	0.80	0.60	0.00	0.00	0.80	0.30	1.00
6	*Atractiella rhizophila*	7.80	3.30	0.30	1.10	3.00	4.80	1.30	1.60	0.30	0.30	0.00	1.10	1.10	0.30	0.00
7	*Bionectria solani*	0.10	0.00	0.00	0.10	0.00	0.10	0.00	0.00	1.70	0.10	0.30	1.00	1.60	1.80	0.00
8	*Cladosporium delicatulum*	41.60	13.30	0.90	3.90	2.40	4.80	39.70	15.70	9.00	21.40	0.80	2.10	8.10	4.90	22.10
9	*Condenascus tortuosus*	0.30	0.20	0.00	0.10	0.50	0.10	0.00	0.30	3.00	1.30	1.80	3.00	1.30	2.40	0.60
10	*Cumuliphoma indica*	0.00	0.10	0.00	0.00	0.10	0.00	0.00	0.00	0.00	6.90	0.00	0.00	0.00	0.00	0.00
11	*Cumuliphoma omnivirens*	0.30	0.40	0.50	2.80	0.30	0.20	0.10	0.90	0.20	14.60	0.50	0.10	5.20	0.40	0.00
12	*Curvularia caricae-papayae*	0.30	0.30	0.00	0.00	0.20	0.00	0.10	7.50	0.10	0.00	0.00	0.00	0.00	0.00	0.00
13	*Cutaneotrichosporon debeurmannianum*	0.40	0.30	0.00	1.00	0.30	0.20	0.20	0.30	1.00	0.50	0.80	0.90	3.50	1.70	0.10
14	*Dictyosporium heptasporum*	0.00	0.00	0.00	4.60	0.10	0.00	0.00	0.00	1.00	0.20	0.00	1.80	0.00	0.60	2.90

15	*Dinemasporium longicapillatum*	0.00	0.00	0.00	0.00	0.00	0.00	0.00	0.00	0.00	0.00	0.00	0.00	0.00	0.00	5.30
16	*Ectophoma multirostrata*	0.00	2.30	0.00	0.00	0.00	0.00	0.00	0.20	1.40	0.90	0.00	0.00	0.00	6.00	0.00
17	*Epicoccum dendrobii*	0.40	1.00	2.20	0.80	0.00	0.90	0.40	2.60	0.00	0.40	0.00	0.00	0.00	0.00	13.50
18	*Epicoccum thailandicum*	7.00	6.70	6.80	8.80	3.40	2.80	8.70	25.10	4.30	4.70	0.10	1.60	3.20	1.60	41.40
19	*Fusarium acutatum*	1.70	2.60	10.50	1.20	34.30	11.80	0.70	3.50	0.80	1.50	3.40	18.70	1.50	2.90	0.40
20	*Fusarium algeriense*	2.00	0.20	0.50	0.90	0.20	0.80	0.40	0.00	0.70	0.20	0.30	0.00	0.00	0.60	0.00
21	*Fusarium kyushuense*	0.90	0.30	0.10	0.10	11.20	0.20	1.70	1.00	2.30	1.40	0.80	6.10	1.90	1.50	0.80
22	*Fusarium ornamentatum*	1.80	0.80	0.10	0.30	0.30	0.00	0.80	4.20	0.00	0.20	0.00	0.00	0.10	0.50	0.00
23	*Gibberella intricans*	1.10	15.10	0.20	1.60	4.70	1.20	2.90	4.10	2.50	4.40	1.30	0.60	4.80	2.50	0.10
24	*Hannaella oryzae*	0.00	0.20	0.00	0.00	0.00	0.00	0.40	0.00	8.90	0.20	0.00	0.10	0.70	0.40	0.50
25	*Memnoniella longistipitata*	0.00	0.00	0.00	0.00	0.00	0.00	0.00	0.00	4.60	2.20	0.30	0.00	0.30	0.00	0.00
26	*Metarhizium majus*	0.30	0.60	0.00	2.50	0.10	0.40	0.00	0.10	0.00	0.10	0.00	0.20	0.40	0.40	0.00
27	*Neocosmospora falciformis*	3.20	11.00	0.10	48.70	2.70	1.50	13.20	2.60	5.00	2.20	3.10	2.20	3.20	5.30	0.20
28	*Neopyrenochaeta spp.*	0.00	0.00	0.00	0.40	0.00	0.00	0.00	0.30	0.60	2.30	1.50	1.90	2.40	0.00	0.00
29	*Nigrospora oryzae*	0.10	1.50	0.20	0.00	0.00	0.80	0.00	0.20	0.00	0.00	0.00	0.00	0.30	0.40	0.00
30	*Oliveonia pauxilla*	0.30	6.90	0.00	0.50	0.00	0.70	0.00	0.00	0.70	0.00	0.00	0.00	0.00	5.80	0.00
31	Others	4.30	6.20	2.10	11.90	5.90	6.30	3.20	3.90	33.40	11.10	18.60	13.40	42.60	28.30	6.60
32	*Papiliotrema laurentii*	0.00	0.00	0.10	0.00	0.10	0.00	0.00	0.00	1.00	0.40	0.30	8.00	1.10	0.40	0.10
33	*Penicillium hetheringtonii*	0.10	0.30	0.00	0.00	0.00	0.20	0.00	1.00	0.40	3.00	0.20	3.50	4.70	0.10	0.00
34	*Periconia macrospinosa*	0.00	0.00	0.00	0.00	0.00	0.00	0.00	0.00	0.00	0.00	0.00	0.00	0.00	0.00	2.70

35	*Poaceascoma spp.*	3.20	6.20	0.50	3.40	8.80	3.50	5.90	1.20	0.70	0.50	0.00	2.60	0.70	0.60	0.00
36	*Preussia flanaganii*	0.00	0.00	0.00	0.00	0.00	0.00	0.00	0.00	3.10	0.10	54.60	0.00	0.40	1.10	0.00
37	*Sarocladium zeae*	0.10	0.00	0.00	0.00	2.90	4.60	0.10	0.20	0.10	0.20	0.00	0.00	0.00	1.90	0.00
38	*Septoria cretae*	0.40	8.70	0.00	0.00	0.00	0.00	0.00	0.00	0.00	0.10	0.00	0.10	0.30	0.00	0.10
39	*Setophoma terrestris*	20.60	10.00	74.40	3.70	16.20	53.60	14.40	16.40	1.70	1.90	0.40	12.40	1.80	23.40	0.10
40	*Stagonosporopsis trachelii*	0.30	0.10	0.00	0.00	0.10	0.00	0.00	1.20	0.60	2.00	0.00	0.00	0.70	0.00	0.00
41	*Westerdykella cylindrica*	0.00	0.00	0.00	0.00	0.00	0.00	0.00	0.00	3.50	0.00	8.60	0.00	3.40	0.30	0.00
	**Total**	**100.00**	**100.00**	**100.00**	**100.00**	**100.00**	**100.00**	**100.00**	**100.00**	**100.00**	**100.00**	**100.00**	**100.00**	**100.00**	**100.00**	**100.00**

R for maize-root samples and S for rhizospheric soil samples; MPP, maize-pigeon pea; MBB, maize-black bean; MCB, maize-common bean; MGG, maize-green gram; and MMC, maize-monoculture cropping systems.

Table S7: Relative abundance (%) of bacterial genera communities in different cropping systems and rhizospheric soil types. Bacterial genera with relative abundances < 1 were grouped as 'other'.

S/No	Genus	Percentage relative abundance				
		**MBB**	**MCB**	**MGG**	**MMC**	**MPP**
1	*Allorhizobium-Neorhizobium-Pararhizobium-Rhizobium*	0.00	5.70	0.00	4.10	2.80
2	*Amycolatopsis*	0.00	0.00	0.00	0.00	0.00
3	*Bacillus*	45.60	51.50	7.10	46.90	26.40
4	*Bradyrhizobium*	19.70	12.70	88.90	21.00	12.70
5	*Burkholderia-Caballeronia-Paraburkholderia*	0.00	0.00	0.00	0.00	3.00
6	*Catenulispora*	0.00	0.00	0.00	0.00	0.00
7	*Dyadobacter*	0.00	0.00	0.10	0.00	1.70
8	*Dyella*	0.00	0.00	0.00	0.00	0.00
9	*Enterobacter*	0.00	0.00	0.00	0.00	0.00
10	*Microvirga*	0.00	2.50	1.30	0.00	13.90
11	*Mitsuaria*	0.00	0.00	0.00	0.00	0.00
12	*Nitrospira*	3.00	4.20	0.40	4.80	3.30
13	Others	11.70	12.50	1.10	16.00	18.50
14	*Pantoea*	0.00	0.00	0.00	0.00	3.20
15	*Pseudarthrobacter*	8.70	8.60	1.00	3.10	2.40
16	*Pseudomonas*	0.00	0.00	0.00	0.00	0.80
17	*Ralstonia*	0.00	0.00	0.00	0.00	0.00
18	*Sphingobium*	3.90	0.00	0.00	3.00	5.00
19	*Sphingomonas*	4.00	2.40	0.10	1.20	2.90
20	*Stenotrophomonas*	0.00	0.00	0.00	0.00	3.30
21	*Streptomyces*	3.40	0.00	0.00	0.00	0.00
	**Total**	**100.00**	**100.00**	**100.00**	**100.00**	**100.00**

MPP, maize-pigeon pea; MBB, maize-black bean; MCB, maize-common bean; MGG, maize-green gram; and MMC, maize-monoculture cropping systems.

Table S8: Relative abundance (%) of bacterial genera communities in different cropping systems and maize-root types. Bacterial genera with relative abundances < 1 were grouped as 'other'.

S/No	Genus	Percentage relative abundance				
		**MBB**	**MCB**	**MGG**	**MMC**	**MPP**
1	*Allorhizobium-Neorhizobium-Pararhizobium-Rhizobium*	0.90	18.70	0.80	1.80	4.10
2	*Amycolatopsis*	1.60	3.80	0.40	2.60	4.20
3	*Bacillus*	7.40	21.30	3.80	0.60	7.10
4	*Bradyrhizobium*	32.10	5.70	74.70	41.00	5.70

5	*Burkholderia-Caballeronia-Paraburkholderia*	1.00	1.90	2.00	11.40	5.00
6	*Catenulispora*	0.00	0.00	0.00	2.00	0.60
7	*Dyadobacter*	0.10	0.40	0.50	0.90	0.70
8	*Dyella*	0.70	0.00	0.40	0.60	0.70
9	*Enterobacter*	0.30	1.20	1.40	0.20	2.60
10	*Microvirga*	0.00	0.00	0.00	0.00	0.00
11	*Mitsuaria*	4.10	12.80	4.40	7.10	20.40
12	*Nitrospira*	0.00	0.00	0.00	0.00	0.00
13	Others	1.40	3.70	2.60	9.00	7.10
14	*Pantoea*	0.50	2.10	1.00	0.60	0.90
15	*Pseudarthrobacter*	0.00	0.80	0.00	0.00	0.00
16	*Pseudomonas*	7.70	15.50	2.20	1.00	7.80
17	*Ralstonia*	37.20	0.50	2.60	15.30	17.90
18	*Sphingobium*	2.50	0.70	1.90	3.20	8.00
19	*Sphingomonas*	0.00	0.40	0.00	1.20	0.70
20	*Stenotrophomonas*	0.20	1.60	0.00	0.50	0.70
21	*Streptomyces*	2.20	8.90	1.30	1.00	5.90
	**Total**	**100.00**	**100.00**	**100.00**	**100.00**	**100.00**

MPP, maize-pigeon pea; MBB, maize-black bean; MCB, maize-common bean; MGG, maize-green gram; and MMC, maize-monoculture cropping systems.

Table S9: Relative abundance (%) of bacterial species communities in study locations, cropping systems, and sample types of fungal species community. Bacterial genera with relative abundances < 1 were grouped as 'other'.

S/No	Genus	Percentage relative abundance														
		**Embu RMCB**	**Embu RMGG**	**Embu RMMC**	**Kitui RMMC**	**Kitui RMPP**	**Meru RMBB**	**Tharaka Nithi RMCB**	**Tharaka Nithi RMGG**	**Embu SMCB**	**Embu SMGG**	**Embu SMMC**	**Kitui SMMC**	**Kitui SMPP**	**Meru SMBB**	**Tharaka Nithi SMCB**
1	*Allorhizobium-Neorhizobium-Pararhizobium-Rhizobium*	4.50	0.60	2.80	1.20	4.10	0.90	59.30	1.10	7.20	0.00	7.90	0.00	2.80	0.00	0.00
2	*Amycolatopsis*	3.90	0.20	4.70	1.40	4.20	1.60	3.60	0.90	0.00	0.00	0.00	0.00	0.00	0.00	0.00
3	*Bacillus*	28.80	3.80	1.70	0.00	7.10	7.40	0.00	3.90	50.50	5.50	52.00	41.30	26.40	45.60	55.30
4	*Bradyrhizobium*	5.10	76.50	7.60	60.00	5.70	32.10	7.50	71.60	12.00	91.50	15.40	26.90	12.70	19.70	15.00
5	*Burkholderia-Caballeronia-Paraburkholderia*	2.40	1.10	9.40	12.60	5.00	1.00	0.70	3.60	0.00	0.00	0.00	0.00	3.00	0.00	0.00
6	*Catenulispora*	0.00	0.00	5.60	0.00	0.60	0.00	0.00	0.00	0.00	0.00	0.00	0.00	0.00	0.00	0.00
7	*Dyadobacter*	0.50	0.00	0.60	1.10	0.70	0.10	0.00	1.30	0.00	0.10	0.00	0.00	1.70	0.00	0.00
8	*Dyella*	0.00	0.60	0.30	0.70	0.70	0.70	0.00	0.00	0.00	0.00	0.00	0.00	0.00	0.00	0.00
9	*Ensifer*	0.00	1.20	0.00	0.90	0.00	0.00	0.00	0.80	0.00	0.00	0.00	0.00	0.00	0.00	0.00
10	*Enterobacter*	1.50	0.30	0.00	0.30	2.60	0.30	0.40	3.30	0.00	0.00	0.00	0.00	0.00	0.00	0.00
11	*Mitsuaria*	14.60	3.60	10.00	5.40	20.40	4.10	7.50	5.80	0.00	0.00	0.00	0.00	0.00	0.00	0.00
12	*Nitrospira*	0.00	0.00	0.00	0.00	0.00	0.00	0.00	0.00	5.40	0.40	2.30	7.50	3.30	3.00	0.00
13	Others	2.90	1.10	11.40	6.80	7.10	1.40	6.00	2.30	15.90	1.40	17.40	14.50	32.40	11.70	11.40
14	*Pantoea*	2.10	0.80	0.50	0.70	0.90	0.50	2.10	1.20	0.00	0.00	0.00	0.00	3.20	0.00	0.00
15	*Pseudarthrobacter*	0.80	0.00	0.00	0.00	0.00	0.00	0.80	0.00	6.20	1.00	0.00	6.40	2.40	8.70	17.30
16	*Pseudomonas*	20.70	3.40	2.00	0.40	7.80	7.70	0.60	0.00	0.00	0.00	0.00	0.00	0.80	0.00	0.00
17	*Ralstonia*	0.70	4.10	31.40	6.00	17.90	37.20	0.00	0.00	0.00	0.00	0.00	0.00	0.00	0.00	0.00
18	*Sphingobium*	0.60	2.00	8.90	0.00	8.00	2.50	1.10	1.70	0.00	0.00	4.10	1.70	5.00	3.90	0.00
19	*Sphingomonas*	0.50	0.00	1.60	0.90	0.70	0.00	0.00	0.00	2.80	0.10	0.70	1.70	2.90	4.00	0.90

20	*Stenotrophomonas*	1.50	0.00	0.00	0.80	0.70	0.20	1.90	0.00	0.00	0.00	0.00	0.00	3.30	0.00	0.00
21	*Streptomyces*	8.90	0.60	1.40	0.70	5.90	2.20	8.60	2.50	0.00	0.00	0.00	0.00	0.00	3.40	0.00
	**Total**	**100.00**	**100.00**	**100.00**	**100.00**	**100.00**	**100.00**	**100.00**	**100.00**	**100.00**	**100.00**	**100.00**	**100.00**	**100.00**	**100.00**	**100.00**

R for maize-root samples and S for rhizospheric soil samples; MPP, maize-pigeon pea; MBB, maize-black bean; MCB, maize-common bean; MGG, maize-green gram; and MMC, maize-monoculture cropping systems.

Table S10: Relative abundance (%) of bacterial species communities in different cropping systems and rhizospheric soil types. Bacterial species with relative abundances < 1 were grouped as 'other'.

S/No	Species	Percentage relative abundance				
		**MBB**	**MCB**	**MGG**	**MMC**	**MPP**
1	*Allorhizobium-Neorhizobium-Pararhizobium-Rhizobium daejeonense*	0.00	0.00	0.00	0.00	0.00
2	*Allorhizobium-Neorhizobium-Pararhizobium-Rhizobium mesosinicum*	0.00	0.00	0.00	0.00	0.00
3	*Allorhizobium-Neorhizobium-Pararhizobium-Rhizobium phaseoli*	0.00	5.70	0.00	4.10	2.80
4	*Amycolatopsis mediterranei*	0.00	0.00	0.00	0.00	0.00
5	*Bacillus aryabhattai*	0.00	2.20	1.00	1.50	3.70
6	*Bacillus drentensis*	15.80	2.50	0.70	4.10	6.20
7	*Bacillus fumarioli*	22.10	21.70	3.20	19.50	14.50
8	*Bacillus megaterium*	0.00	8.00	0.00	12.40	0.00
9	*Bacillus nealsonii*	7.70	14.90	1.90	2.80	2.00
10	*Bacillus pseudofirmus*	0.00	0.00	0.00	0.00	0.00
11	*Bradyrhizobium elkanii*	19.70	11.10	88.90	20.10	8.90
12	*Bradyrhizobium japonicum*	0.00	0.00	0.00	0.00	0.00
13	*Bradyrhizobium liaoningense*	0.00	1.60	0.00	0.90	0.00
14	*Bradyrhizobium yuanmingense*	0.00	0.00	0.00	0.00	3.90
15	*Burkholderia-Caballeronia-Paraburkholderia cenocepacia*	0.00	0.00	0.00	0.00	0.00
16	*Burkholderia-Caballeronia-Paraburkholderia contaminans*	0.00	0.00	0.00	0.00	0.00
17	*Burkholderia-Caballeronia-Paraburkholderia gladioli*	0.00	0.00	0.00	0.00	0.00
18	*Burkholderia-Caballeronia-Paraburkholderia lata*	0.00	0.00	0.00	0.00	0.00
19	*Burkholderia-Caballeronia-Paraburkholderia phenoliruptrix*	0.00	0.00	0.00	0.00	0.00
20	*Catenulispora acidiphila*	0.00	0.00	0.00	0.00	0.00
21	*Dyadobacter fermentans*	0.00	0.00	0.10	0.00	1.70
22	*Dyella marensis*	0.00	0.00	0.00	0.00	0.00
23	*Ensifer fredii*	0.00	0.00	0.00	0.00	0.00
24	*Enterobacter roggenkampii*	0.00	0.00	0.00	0.00	0.00
25	*Microvirga flocculans*	0.00	2.50	1.30	0.00	13.90
26	*Mitsuaria chitosanitabida*	0.00	0.00	0.00	0.00	0.00
27	*Nitrospira japonica*	3.00	4.20	0.40	4.80	3.30
28	*Novosphingobium aromaticivorans*	0.00	0.00	0.00	0.00	0.00
29	Others	19.50	17.10	1.60	23.80	25.20

30	*Pantoea dispersa*	0.00	0.00	0.00	0.00	3.20
31	*Pseudarthrobacter sulfonivorans*	4.90	8.60	0.80	3.10	2.40
32	*Pseudomonas psychrotolerans*	0.00	0.00	0.00	0.00	0.00
33	*Pseudomonas stutzeri*	0.00	0.00	0.00	0.00	0.00
34	*Ralstonia pickettii*	0.00	0.00	0.00	0.00	0.00
35	*Sphingobium fuliginis*	3.90	0.00	0.00	3.00	5.00
36	*Sphingomonas leidyi*	0.00	0.00	0.00	0.00	0.00
37	*Stenotrophomonas maltophilia*	0.00	0.00	0.00	0.00	3.30
38	*Streptomyces griseorubiginosus*	3.40	0.00	0.00	0.00	0.00
39	*Streptomyces lincolnensis*	0.00	0.00	0.00	0.00	0.00
40	*Streptomyces scabiei*	0.00	0.00	0.00	0.00	0.00
41	*Variovorax paradoxus*	0.00	0.00	0.00	0.00	0.00
	**Total**	**100.00**	**100.00**	**100.00**	**100.00**	**100.00**

MPP, maize-pigeon pea; MBB, maize-black bean; MCB, maize-common bean; MGG, maize-green gram; and MMC, maize-monoculture cropping systems.

Table S11: Relative abundance (%) of bacterial species communities in different cropping systems and maize-root types. Bacterial species with relative abundances < 1 were grouped as 'other'.

S/No	Species	Percentage relative abundance				
		**MBB**	**MCB**	**MGG**	**MMC**	**MPP**
1	*Allorhizobium-Neorhizobium-Pararhizobium-Rhizobium daejeonense*	0.00	1.20	0.00	0.10	1.00
2	*Allorhizobium-Neorhizobium-Pararhizobium-Rhizobium mesosinicum*	0.00	1.10	0.30	0.00	0.90
3	*Allorhizobium-Neorhizobium-Pararhizobium-Rhizobium phaseoli*	0.90	16.10	0.50	1.60	2.20
4	*Amycolatopsis mediterranei*	1.60	3.80	0.40	2.60	4.20
5	*Bacillus aryabhattai*	0.40	2.60	1.00	0.00	0.50
6	*Bacillus drentensis*	0.00	0.00	0.00	0.30	0.00
7	*Bacillus fumarioli*	0.00	0.00	0.00	0.30	0.00
8	*Bacillus megaterium*	0.50	1.90	0.90	0.00	0.70
9	*Bacillus nealsonii*	0.00	0.00	0.00	0.00	0.00
10	*Bacillus pseudofirmus*	6.50	16.30	1.90	0.00	6.00
11	*Bradyrhizobium elkanii*	28.90	3.20	7.00	3.00	1.60
12	*Bradyrhizobium japonicum*	0.10	0.50	0.10	0.10	0.80
13	*Bradyrhizobium liaoningense*	0.40	0.40	0.10	0.20	0.40
14	*Bradyrhizobium yuanmingense*	2.70	1.60	67.40	37.60	2.80
15	*Burkholderia-Caballeronia-Paraburkholderia cenocepacia*	0.00	0.20	0.00	0.20	1.70
16	*Burkholderia-Caballeronia-Paraburkholderia contaminans*	0.50	0.00	0.10	0.00	1.30

17	*Burkholderia-Caballeronia-Paraburkholderia gladioli*	0.00	1.30	1.30	9.50	0.00
18	*Burkholderia-Caballeronia-Paraburkholderia lata*	0.10	0.50	0.40	0.80	0.00
19	*Burkholderia-Caballeronia-Paraburkholderia phenoliruptrix*	0.50	0.00	0.10	0.70	1.50
20	*Catenulispora acidiphila*	0.00	0.00	0.00	2.00	0.60
21	*Dyadobacter fermentans*	0.10	0.40	0.50	0.90	0.70
22	*Dyella marensis*	0.70	0.00	0.40	0.60	0.70
23	*Ensifer fredii*	0.00	0.00	0.80	0.60	0.00
24	*Enterobacter roggenkampii*	0.30	1.20	1.20	0.20	2.60
25	*Microvirga flocculans*	0.00	0.00	0.00	0.00	0.00
26	*Mitsuaria chitosanitabida*	4.10	12.80	4.40	7.10	20.40
27	*Nitrospira japonica*	0.00	0.00	0.00	0.00	0.00
28	*Novosphingobium aromaticivorans*	0.00	0.30	0.50	0.00	0.60
29	Others	2.10	4.70	1.70	8.30	6.60
30	*Pantoea dispersa*	0.50	2.10	1.00	0.60	0.90
31	*Pseudarthrobacter sulfonivorans*	0.00	0.80	0.00	0.00	0.00
32	*Pseudomonas psychrotolerans*	0.00	0.20	0.20	0.30	1.30
33	*Pseudomonas stutzeri*	7.70	15.20	2.00	0.00	6.40
34	*Ralstonia pickettii*	37.20	0.50	2.60	15.30	17.90
35	*Sphingobium fuliginis*	2.50	0.70	1.90	3.20	8.00
36	*Sphingomonas leidyi*	0.00	0.40	0.00	1.10	0.70
37	*Stenotrophomonas maltophilia*	0.10	1.40	0.00	0.50	0.70
38	*Streptomyces griseorubiginosus*	1.20	7.10	0.80	0.50	5.00
39	*Streptomyces lincolnensis*	0.20	0.50	0.50	0.00	0.40
40	*Streptomyces scabiei*	0.00	1.10	0.00	0.00	0.50
41	*Variovorax paradoxus*	0.20	0.00	0.00	1.60	0.30
	**Total**	**100.00**	**100.00**	**100.00**	**100.00**	**100.00**

MPP, maize-pigeon pea; MBB, maize-black bean; MCB, maize-common bean; MGG, maize-green gram; and MMC, maize-monoculture cropping systems.

Table S12: Relative abundance (%) of bacterial species communities in study locations, cropping systems and sample types. Bacterial species with relative abundances < 1 were grouped as 'other'.

S/No	Species	Percentage relative abundance														
		**Embu RMCB**	**Embu RMGG**	**Embu RMMC**	**Kitui RMMC**	**Kitui RMPP**	**Meru RMBB**	**Tharaka Nithi RMCB**	**Tharaka Nithi RMGG**	**Embu SMCB**	**Embu SMGG**	**Embu SMMC**	**Kitui SMMC**	**Kitui SMPP**	**Meru SMBB**	**Tharaka Nithi SMCB**
1	*Allorhizobium-Neorhizobium-Pararhizobium-Rhizobium daejeonense*	1.20	0.00	0.00	0.10	1.00	0.00	1.10	0.00	0.00	0.00	0.00	0.00	0.00	0.00	0.00
2	*Allorhizobium-Neorhizobium-Pararhizobium-Rhizobium mesosinicum*	0.80	0.00	0.00	0.00	0.90	0.00	2.00	0.70	0.00	0.00	0.00	0.00	0.00	0.00	0.00
3	*Allorhizobium-Neorhizobium-Pararhizobium-Rhizobium phaseoli*	2.50	0.60	2.70	1.00	2.20	0.90	55.10	0.40	7.20	0.00	7.90	0.00	2.80	0.00	0.00
4	*Amycolatopsis mediterranei*	3.90	0.20	4.70	1.40	4.20	1.60	3.60	0.90	0.00	0.00	0.00	0.00	0.00	0.00	0.00
5	*Bacillus aryabhattai*	3.60	0.50	0.00	0.00	0.50	0.40	0.00	1.90	0.00	1.10	3.00	0.00	3.70	0.00	10.30
6	*Bacillus drentensis*	0.00	0.00	0.80	0.00	0.00	0.00	0.00	0.00	2.20	0.70	5.20	3.00	6.20	15.80	3.60
7	*Bacillus fumarioli*	0.00	0.00	0.90	0.00	0.00	0.00	0.00	0.00	20.60	1.90	23.60	15.00	14.50	22.10	25.70
8	*Bacillus megaterium*	2.50	0.40	0.00	0.00	0.70	0.50	0.00	1.80	10.20	0.00	10.30	14.60	0.00	0.00	0.00
9	*Bacillus nealsonii*	0.00	0.00	0.00	0.00	0.00	0.00	0.00	0.00	14.70	1.50	5.40	0.00	2.00	7.70	15.80
10	*Bacillus pseudofirmus*	22.00	2.90	0.00	0.00	6.00	6.50	0.00	0.00	0.00	0.00	0.00	0.00	0.00	0.00	0.00

11	*Bradyrhizobium elkanii*	3.30	4.40	6.70	0.90	1.60	28.90	3.10	11.80	10.00	91.50	15.40	25.10	8.90	19.70	15.00
12	*Bradyrhizobium liaoningense*	0.30	0.00	0.60	0.00	0.40	0.40	0.70	0.40	2.00	0.00	0.00	1.80	0.00	0.00	0.00
13	*Bradyrhizobium yuanmingense*	0.90	72.00	0.00	59.10	2.80	2.70	3.40	59.20	0.00	0.00	0.00	0.00	3.90	0.00	0.00
14	*Burkholderia-Caballeronia-Paraburkholderia cenocepacia*	0.20	0.00	0.60	0.00	1.70	0.00	0.10	0.00	0.00	0.00	0.00	0.00	0.00	0.00	0.00
15	*Burkholderia-Caballeronia-Paraburkholderia contaminans*	0.00	0.20	0.00	0.00	1.30	0.50	0.00	0.00	0.00	0.00	0.00	0.00	0.00	0.00	0.00
16	*Burkholderia-Caballeronia-Paraburkholderia gladioli*	1.70	0.00	4.00	12.60	0.00	0.00	0.00	3.60	0.00	0.00	0.00	0.00	0.00	0.00	0.00
17	*Burkholderia-Caballeronia-Paraburkholderia lata*	0.40	0.70	2.30	0.00	0.00	0.10	0.50	0.00	0.00	0.00	0.00	0.00	0.00	0.00	0.00
18	*Burkholderia-Caballeronia-Paraburkholderia phenoliruptrix*	0.00	0.20	2.10	0.00	1.50	0.50	0.00	0.00	0.00	0.00	0.00	0.00	0.00	0.00	0.00
19	*Catenulispora acidiphila*	0.00	0.00	5.60	0.00	0.60	0.00	0.00	0.00	0.00	0.00	0.00	0.00	0.00	0.00	0.00
20	*Dyadobacter fermentans*	0.50	0.00	0.60	1.10	0.70	0.10	0.00	1.30	0.00	0.10	0.00	0.00	1.70	0.00	0.00
21	*Dyella marensis*	0.00	0.60	0.30	0.70	0.70	0.70	0.00	0.00	0.00	0.00	0.00	0.00	0.00	0.00	0.00
22	*Ensifer fredii*	0.00	1.20	0.00	0.90	0.00	0.00	0.00	0.00	0.00	0.00	0.00	0.00	0.00	0.00	0.00

23	*Enterobacter roggenkampii*	1.50	0.00	0.00	0.30	2.60	0.30	0.40	3.30	0.00	0.00	0.00	0.00	0.00	0.00	0.00
24	*Mesorhizobium plurifarium*	0.00	0.50	0.00	0.00	0.30	0.00	1.10	0.40	0.00	0.00	0.00	0.00	1.80	0.00	0.00
25	*Microvirga flocculans*	0.00	0.00	0.00	0.00	0.00	0.00	0.00	0.00	3.20	0.20	0.00	0.00	13.90	0.00	0.00
26	*Mitsuaria chitosanitabida*	14.60	3.60	10.00	5.40	20.40	4.10	7.50	5.80	0.00	0.00	0.00	0.00	0.00	0.00	0.00
27	*Nitrospira japonica*	0.00	0.00	0.00	0.00	0.00	0.00	0.00	0.00	5.40	0.40	2.30	7.50	3.30	3.00	0.00
28	*Novosphingobium aromaticivorans*	0.40	0.00	0.00	0.00	0.60	0.00	0.00	1.40	0.00	0.00	0.00	0.00	0.00	0.00	0.00
29	Others	4.40	1.10	12.60	6.00	7.10	2.30	6.50	1.80	18.30	1.70	22.70	24.90	23.30	19.50	12.40
30	*Pantoea dispersa*	2.10	0.80	0.50	0.70	0.90	0.50	2.10	1.20	0.00	0.00	0.00	0.00	3.20	0.00	0.00
31	*Pseudarthrobacter sulfonivorans*	0.80	0.00	0.00	0.00	0.00	0.00	0.80	0.00	6.20	0.90	0.00	6.40	2.40	4.90	17.30
32	*Pseudomonas psychrotolerans*	0.00	0.30	0.00	0.40	1.30	0.00	0.60	0.00	0.00	0.00	0.00	0.00	0.00	0.00	0.00
33	*Pseudomonas stutzeri*	20.50	3.10	0.00	0.00	6.40	7.70	0.00	0.00	0.00	0.00	0.00	0.00	0.00	0.00	0.00
34	*Ralstonia pickettii*	0.70	4.10	31.40	6.00	17.90	37.20	0.00	0.00	0.00	0.00	0.00	0.00	0.00	0.00	0.00
35	*Sphingobium fuliginis*	0.60	2.00	8.90	0.00	8.00	2.50	1.10	1.70	0.00	0.00	4.10	1.70	5.00	3.90	0.00
36	*Sphingomonas leidyi*	0.50	0.00	1.40	0.90	0.70	0.00	0.00	0.00	0.00	0.00	0.00	0.00	0.00	0.00	0.00
37	*Stenotrophomonas maltophilia*	1.40	0.00	0.00	0.80	0.70	0.10	1.50	0.00	0.00	0.00	0.00	0.00	3.30	0.00	0.00
38	*Streptomyces griseorubiginosus*	7.40	0.60	1.40	0.00	5.00	1.20	6.10	1.00	0.00	0.00	0.00	0.00	0.00	3.40	0.00
39	*Streptomyces lincolnensis*	0.50	0.00	0.00	0.00	0.40	0.20	0.50	1.40	0.00	0.00	0.00	0.00	0.00	0.00	0.00
40	*Streptomyces scabiei*	0.80	0.00	0.00	0.00	0.50	0.00	2.00	0.00	0.00	0.00	0.00	0.00	0.00	0.00	0.00

41	*Variovorax paradoxus*	0.00	0.00	1.80	1.50	0.30	0.20	0.00	0.00	0.00	0.00	0.00	0.00	0.00	0.00	0.00
	**Total**	**100.00**	**100.00**	**100.00**	**100.00**	**100.00**	**100.00**	**100.00**	**100.00**	**100.00**	**100.00**	**100.00**	**100.00**	**100.00**	**100.00**	**100.00**

R for maize-root samples and S for rhizospheric soil samples; MPP, maize-pigeon pea; MBB, maize-black bean; MCB, maize-common bean; MGG, maize-green gram; and MMC, maize-monoculture cropping systems.

Table S13: Alpha diversity of fungal species communities based on different cropping systems.

Cropping systems		
**Samples name**	***Chao1 estimator P* value**	***Shannon estimator P* value**
MBB vs MCB	0.73	0.47
MBB vs MGG	0.54	0.54
MBB vs MMC	0.73	0.84
MBB vs MPP	0.16	0.38
MCB vs MGG	0.69	0.17
MCB vs MMC	0.59	0.56
MCB vs MPP	0.35	0.13
MGG vs MMC	0.95	0.85
MGG vs MPP	0.38	0.69
MMC vs MPP	0.22	0.37

MPP, maize-pigeon pea; MBB, maize-black bean; MCB, maize-common bean; MGG, maize-green gram; and MMC, maize-monoculture cropping systems.

Table S14: Alpha diversity of fungal species communities based on study locations.

Study locations		
**Samples name**	***Chao1 estimator P* value**	***Shannon estimator P* value**
Embu vs Kitui	0.17	0.11
Embu vs Meru	0.75	0.85
Embu vs Tharaka Nithi	1.00	0.76
Kitui vs Meru	0.26	0.31
Kitui vs Tharaka Nithi	0.22	0.19
Meru vs Tharaka Nithi	1.00	0.53

Table S15: Alpha diversity of fungal species communities based on different cropping systems and sample types.

**Samples name**	**Cropping systems and sample types**			
	***Chao1 estimator P* value**		***Shannon estimator P* value**	
	**Maize-root**	**Rhizospheric soil**	**Maize-root**	**Rhizospheric soil**
RMBB vs RMCB	0.55	0.86	0.69	0.38
RMBB vs RMGG	0.05*	0.20	0.15	0.40
RMBB vs RMMC	1.00	0.27	0.86	0.27
RMBB vs RMPP	0.26	0.80	0.41	1.00
RMCB vs RMGG	0.22	0.57	0.09	1.00
RMCB vs RMMC	1.00	0.02*	0.86	0.12
RMCB vs RMPP	0.39	0.79	0.56	0.03*
RMGG vs RMMC	0.39	0.13	0.09	0.73
RMGG vs RMPP	1.00	0.10	0.56	0.10
RMMC vs RMPP	0.48	0.23	0.53	0.11

R for maize-root samples and S for rhizospheric soil samples; MPP, maize-pigeon pea; MBB, maize-black bean; MCB, maize-common bean; MGG, maize-green gram; and MMC, maize-monoculture cropping system. *Indicated significant effects.

Table S16: Alpha diversity of fungal species communities based on different cropping systems and study locations.

**Cropping systems and study locations**		
**Samples name**	***Chao1 estimator P* value**	***Shannon estimator P* value**
EMCB vs EMGG	0.75	0.49
EMCB vs EMMC	0.84	0.78
EMCB vs KMMC	0.63	0.28
EMCB vs KMPP	0.60	0.23
EMCB vs MMBB	0.61	0.78
EMCB vs TMCB	0.43	0.71
EMCB vs TMGG	0.53	0.71
EMGG vs EMMC	0.90	0.38
EMGG vs KMMC	0.38	0.71
EMGG vs KMPP	0.28	0.73
EMGG vs MMBB	0.95	0.53
EMGG vs TMCB	0.43	0.07
EMGG vs KMGG	0.14	1.00
EMMC vs KMMC	0.40	0.20
EMMC vs KMPP	0.25	0.18
EMMC vs MMBB	0.40	0.27
EMMC vs TMCB	0.80	0.80
EMMC vs KMGG	0.40	0.80
KMMC vs KMPP	0.25	1.00
KMMC vs MMBB	0.52	0.52
KMMC vs TMCB	0.20	0.80
KMMC vs TMGG	0.40	0.80
KMPP vs MMBB	0.16	0.38
KMPP vs MMCB	0.19	0.22
KMPP vs TMGG	1.00	0.89
MMBB vs TMCB	0.89	0.22
MMBB vs TMGG	0.22	1.00
TMCB vs TMGG	0.33	0.33

MPP, maize-pigeon pea; MBB, maize-black bean; MCB, maize-common bean; MGG, maize-green; and MMC, maize-monoculture cropping systems. E, Embu; M, Meru; T, Tharaka Nithi; K, Kitui.

Table S17: Alpha diversity of bacterial species communities based on different cropping systems.

**Cropping systems**

Samples name	*Chao1 estimator P* value	*Shannon estimator P* value
MBB vs MCB	0.81	0.96
MBB vs MGG	0.14	0.21
MBB vs MMC	0.68	0.38
MBB vs MPP	0.38	0.40
MCB vs MGG	0.36	0.55
MCB vs MMC	0.39	0.48
MCB vs MPP	0.31	0.51
MGG vs MMC	0.78	0.97
MGG vs MPP	0.98	0.74
MMC vs MPP	0.96	0.97

MPP, maize-pigeon pea; MBB, maize-black bean; MCB, maize-common bean; MGG, maize-green gram; and MMC, maize-monoculture cropping systems.

Table S18: Alpha diversity of bacterial species communities based on study locations.

Study locations		
**Samples name**	***Chao1 estimator P* value**	***Shannon estimator P* value**
Embu vs Kitui	0.65	0.72
Embu vs Meru	0.76	0.61
Embu vs Tharaka Nithi	0.72	0.62
Kitui vs Meru	0.40	0.40
Kitui vs Tharaka Nithi	0.96	0.96
Meru vs Tharaka Nithi	0.19	0.20

Table S19: Alpha diversity of bacterial species communities based on the different cropping systems and sample types.

Samples name	Cropping systems and sample types			
	***Chao1 estimator P* value**		***Shannon estimator P* value**	
	**Maize-root**	**Rhizospheric soil**	**Maize-root**	**Rhizospheric soil**
RMBB vs RMCB	0.09	0.55	0.42	0.19
RMBB vs RMGG	0.15	0.48	0.09	0.80
RMBB vs RMMC	0.92	0.33	0.35	0.38
RMBB vs RMPP	1.00	0.30	1.00	0.63
RMCB vs RMGG	0.03*	0.41	0.22	0.29
RMCB vs RMMC	0.05*	0.69	0.22	0.65
RMCB vs RMPP	0.11	0.33	0.09	0.11
RMGG vs RMMC	0.05*	0.73	0.22	0.73
RMGG vs RMPP	0.11	0.27	0.90	0.20
RMMC vs RMPP	0.05*	0.71	0.41	0.56

*Indicated significant effects. R, maize-root samples; MPP, maize-pigeon pea; MBB, maize-black bean; MCB, maize-common bean; MGG, maize-green gram; and MMC, maize-monoculture cropping systems.

Table S20: Alpha diversity of bacterial species communities based on cropping systems and study locations.

Cropping systems and study locations		
**Samples name**	***Chao1 estimator P* value**	***Shannon estimator P* value**
EMCB vs EMGG	0.49	0.41
EMCB vs EMMC	0.34	0.34
EMCB vs KMMC	0.68	0.68
EMCB vs KMPP	0.27	0.38
EMCB vs MMBB	0.60	0.68
EMCB vs TMCB	0.89	0.71
EMCB vs TMGG	0.50	0.50
EMGG vs EMMC	0.94	0.94
EMGG vs KMMC	0.91	1.00
EMGG vs KMPP	0.95	0.75
EMGG vs MMBB	0.28	0.37
EMGG vs TMCB	0.86	0.64
EMGG vs KMGG	0.90	1.00
EMMC vs KMMC	0.91	0.76
EMMC vs KMPP	0.95	0.75
EMMC vs MMBB	0.72	0.37
EMMC vs TMCB	0.86	1.00
EMMC vs KMGG	0.71	1.00
KMMC vs KMPP	1.00	0.81
KMMC vs MMBB	0.70	0.70
KMMC vs TMCB	1.00	0.80
KMMC vs TMGG	0.17	1.00
KMPP vs MMBB	0.38	0.40
KMPP vs TMCB	0.90	0.89
KMPP vs TMGG	0.76	0.92
MMBB vs TMCB	0.66	0.50
MMBB vs TMGG	0.17	0.27
TMCB vs TMGG	0.80	0.80

MPP, maize-pigeon pea; MBB, maize-black bean; MCB, maize-common bean; MGG, maize-green gram; and MMC, maize-monoculture cropping system. E, Embu; M, Meru; T, Tharaka Nithi; K, Kitui.

Table S21: Results of PERMANOVA testing the effects and interaction between different cropping systems, sample types, and study locations on fungal communities from smallholder farms.

**Factor**	***df***	**Sum of Sqs**	R^2	***F* value**	***P* value**
Cropping systems	4	2.081	0.104	1.135	0.080

Residual	39	17.878	0.896	*NA*	*NA*
Cropping systems and sample types	9	4.502	0.364	1.784	**0.0001**
Residual	28	7.851	0.636	*NA*	*NA*
Cropping systems and study locations	7	3.165	0.256	1.476	**0.0001**
Residual	30	9.188	0.744	*NA*	*NA*
Study locations	3	1.693	0.137	1.800	**0.0001**
Residual	34	10.659	0.863	*NA*	*NA*

Key: *NA*, Negligible. Significant effects are indicated in bold at $P < 0.05$.

Table S22: Results of PERMANOVA testing the effects and interaction between the different cropping systems, sample types, and study location in bacterial species communities from smallholder farms.

**Factor**	***df***	**Sum of Sqs**	R^2	***F* value**	***P* value**
Cropping systems	4	1.958	0.097	1.054	0.262
Residual	39	18.103	0.902	*NA*	*NA*
Cropping systems and sample types	9	5.169	0.257	1.311	**0.0001**
Residual	34	14.891	0.742	*NA*	*NA*
Cropping systems and study locations	7	3.027	0.151	0.914	0.910
Residual	36	16.889	0.846	*NA*	*NA*
Study Locations	3	1.110	0.055	0.781	0.995
Residual	40	18.950	0.944	*NA*	*NA*

Key: *NA*, Negligible. Significant effects are indicated in bold at $P < 0.05$.
